# Rectus sheath catheters *versus* thoracic epidural analgesia for pain management after open surgery: systematic review and meta-analysis

**DOI:** 10.1093/bjs/znag058

**Published:** 2026-05-13

**Authors:** Christian D Fankhauser, Stefan Breitenstein, Hans Gelpke, Caveh Madjdpour, Gesine Meili, Leila Sultan-Beyer, Thomas R Wyss, Ernest Kaufmann

**Affiliations:** Division of Urology, Department of Surgery, Cantonal Hospital of Winterthur, Winterthur, Switzerland; Faculty of Health Sciences and Medicine, University of Lucerne, Lucerne, Switzerland; Clinic for Urology, University Teaching and Research Hospital of the University of Lucerne, Lucerne, Switzerland; Faculty of Medicine, University of Zurich, Zurich, Switzerland; Faculty of Medicine, University of Zurich, Zurich, Switzerland; Department of Surgery, Cantonal Hospital of Winterthur, Winterthur, Switzerland; Department of Surgery, Cantonal Hospital of Winterthur, Winterthur, Switzerland; Faculty of Medicine, University of Zurich, Zurich, Switzerland; Department of Anaesthesiology, Cantonal Hospital of Winterthur, Winterthur, Switzerland; Department of Gynaecology and Obstetrics, Cantonal Hospital of Winterthur, Winterthur, Switzerland; Department of Gynaecology and Obstetrics, Cantonal Hospital of Winterthur, Winterthur, Switzerland; Department of Vascular Surgery, Inselspital, Bern University Hospital, University of Bern, Bern, Switzerland; Department of Interventional Radiology and Vascular Surgery, Cantonal Hospital of Winterthur, Winterthur, Switzerland; Faculty of Health Sciences and Medicine, University of Lucerne, Lucerne, Switzerland; Clinic for Urology, University Teaching and Research Hospital of the University of Lucerne, Lucerne, Switzerland

## Abstract

**Background:**

Thoracic epidural analgesia (TEA) remains the ‘gold standard’ for postoperative pain management after major open surgery, but is potentially associated with hypotension, urinary retention, and delayed recovery. Rectus sheath catheters (RSCs) offer a simple regional alternative that avoids sympathetic blockade while maintaining somatic analgesia. The aim of this review was to compare analgesic efficacy, complications, recovery, patient satisfaction, and costs between RSCs and TEA in open surgical procedures.

**Methods:**

This systematic review was registered with PROSPERO, the international prospective register of systematic reviews (registration number: CRD420251234467). A systematic PubMed search was conducted to identify studies comparing continuous wound infusion via RSCs with TEA in adult patients undergoing open abdominal, pelvic, thoracic, or vascular surgery. RCTs, as well as prospective and retrospective comparative studies, were included. A meta-analysis was performed for randomized trials.

**Results:**

In total, 31 studies (21 prospective and 10 retrospective) involving 2162 patients were included. RSCs and TEA did not differ significantly with respect to postoperative pain (standardized mean difference −0.35 (95% c.i. −2.01 to 1.32)) or opioid consumption (standardized mean difference −0.32 (95% c.i. −1.71 to 1.07)). No differences were observed in recovery of bowel function, urinary retention, time to mobilization, or length of hospital stay. RSCs significantly reduced the risk of hypotension compared with TEA (risk ratio 0.40 (95% c.i. 0.26 to 0.60)) and were associated with lower costs with savings ranging from $500 to $6632 per case. Subgroup analyses suggested less urinary retention and earlier mobilization with RSCs in non-visceral surgery and non-laparotomy incisions.

**Conclusion:**

RSCs provide analgesia comparable to TEA with fewer complications, facilitating earlier recovery and potential cost savings. Considering the growing shift toward fast-track surgery, RSCs represent a pragmatic and resource-efficient alternative for postoperative pain management in open surgical procedures.

## Teaser

This systematic review and meta-analysis compared rectus sheath catheters (RSCs) with thoracic epidural analgesia (TEA) for postoperative pain management after open surgery. Across 31 studies including 2162 patients, RSCs provided analgesia and opioid requirements comparable to TEA, with no differences in recovery outcomes, while significantly reducing hypotension and overall costs. RSCs represent a safe, cost-effective alternative to TEA that may support enhanced recovery pathways in open surgical procedures.

## Introduction

Effective analgesia remains a cornerstone of enhanced recovery after surgery (ERAS) protocols^1^. Thoracic epidural analgesia (TEA) is considered the ‘gold standard’ for open procedures, providing excellent pain control, but carrying the risk of hypotension, urinary retention, and delayed mobilization^2^. Rectus sheath catheters (RSCs), which deliver a continuous infusion of local anaesthetic within the rectus sheath or preperitoneal space, have emerged as a simple, potentially safer alternative in several surgical disciplines^3,4^, especially when TEA is contraindicated. This regional technique avoids sympathetic blockade while maintaining effective somatic analgesia. However, evidence comparing RSCs and TEA remains heterogeneous across surgical disciplines, incision types, and study designs. The aim of this review was to systematically summarize and compare available data from randomized and non-randomized studies evaluating RSCs *versus* TEA for open surgical procedures, focusing on analgesic efficacy, opioid consumption, complications, functional recovery, patient satisfaction, and costs.

## Methods

A systematic literature search was conducted on PubMed on 3 November 2025 in accordance with the PRISMA statement^5^ to identify studies directly comparing continuous wound infusion via RSCs with TEA in patients undergoing open surgery. This systematic review was registered with PROSPERO, the international prospective register of systematic reviews (registration number: CRD420251234467).

RCTs, prospective comparative studies, and retrospective comparative cohort studies were eligible for inclusion in the systematic review. Although the PROSPERO registration initially specified inclusion of RCTs only, the search strategy was intentionally broad to ensure that no relevant RCTs were missed. During the screening stage, observational studies were identified and subsequently included as supplementary evidence, but only RCTs were pooled in the meta-analysis. Non-English literature, publications before 1990, reviews, case reports, purely laparoscopic studies, studies including paediatric populations, and non-comparative series were excluded. Reference lists of identified publications were manually screened to find additional relevant studies, and EndNote’s ‘close match’ function was used to filter out duplicate articles. Two investigators (E.K. and C.D.F.) screened all titles, abstracts, and full texts for inclusion in the study. Data from the same study that appeared in multiple publications were only included once. Finally, only published articles containing a minimum of 20 patients in the analysis were included. Any discrepancies were resolved by a third investigator (S.B.). Further details on the complete search strategy and inclusion criteria are provided in *Appendices S1*, *S2*.

Risk of bias was assessed for all randomized trials across standard Cochrane domains using the Cochrane risk-of-bias tool RoB 1 and is summarized graphically. Outcomes were organized into five thematic domains: pain and opioid consumption; complications (hypotension, bowel function, urinary retention, and local analgesia catheter issues); recovery and length of stay; patient satisfaction; and costs. Details on outcome definitions are summarized in *Appendix S1*. To demonstrate the direction of outcomes across heterogeneous study designs, a qualitative heatmap was generated. Outcomes favouring RSCs are shown in green, those favouring TEA are shown in red, non-significant or similar results are shown in blue, and unreported outcomes are shown in white.

A formal meta-analysis was subsequently performed exclusively on RCTs to derive pooled effect estimates based on the highest level of evidence. For continuous outcomes measured on different scales, standardized mean differences (SMDs) and corresponding standard errors were calculated using pooled standard deviations. Both fixed-effect and random-effects models were applied. Effect estimates were pooled using SMDs or risk ratios (RRs) with 95% confidence intervals. Random-effects models were pre-specified as primary owing to anticipated heterogeneity; fixed-effect models were applied when heterogeneity was low. Subgroup analyses were also performed, stratified by surgical category and incision type to explore and account for anticipated clinical heterogeneity across the included studies.

Sensitivity analyses were performed using leave-one-out approaches and influence diagnostics. Exploratory meta-regression analyses were conducted for outcomes with a sufficient number of included studies to allow exploratory assessment, evaluating surgical category, incision type, and publication year.

All statistical tests were two-sided, and *P* < 0.050 was considered statistically significant.

Statistical analyses were performed using R Studio (R Core Team, 2022, version 2023.03.0+386). Further statistical details are provided in *Appendix S1*.

## Results

**Fig. 1:**
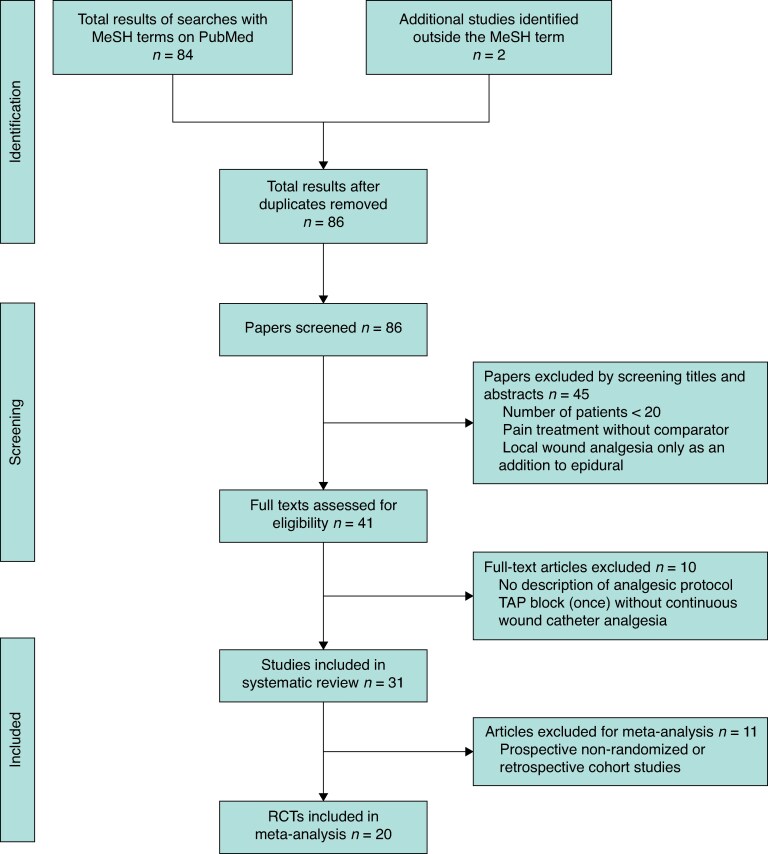
PRISMA diagram showing selection of the included studies MeSH, medical subject headings, TAP, transversus abdominis plane.

The initial search identified 86 publications, and, after title and abstract screening, 41 full texts were assessed for eligibility. After assessment, 31 studies were included in the analysis, of which 21 were prospective and 10 were retrospective (*Fig. 1* and *Appendix S3*). These 31 studies included a range of 12–141 patients, providing data for a total of 2162 patients. Of these, 20 RCTs (30–134 patients per study with a total of 1368 patients) were identified and included in the meta-analysis.

The included studies covered a broad range of predominantly abdominal surgical specialties, including visceral, urological, gynaecological, and vascular surgery; only one study involved thoracic procedures (*Appendix S3*). Procedures were predominantly performed through midline, subcostal, or Pfannenstiel incisions, with some cases involving thoracotomy or transverse abdominal approaches. RSCs were typically placed bilaterally under direct vision or ultrasonographic guidance in the preperitoneal space before wound closure, yet the description of the exact catheter position varied across studies, including subfascial, preperitoneal, transversus abdominis plane, rectus sheath, suprafascial, and positioning along the inferior border of the ribs. The most commonly used continuous infusions were (levo)bupivacaine 0.125–0.25% or ropivacaine 0.1–0.375%. Infusion rates ranged from 2 to 12 ml/h divided across both catheters, with bolus doses of 5–10 ml administered intermittently in some protocols. Epidural groups received thoracic epidural infusions containing bupivacaine 0.125–0.25% or ropivacaine 0.1–0.2% alone, or mostly in combination with fentanyl (2 µg/ml), morphine (0.02–0.1 mg/ml), or sufentanil (0.4–1 µg/ml), with infusion rates ranging from 5 to 10 ml/h. Reported infusion durations for both groups ranged from 2 to 5 days, depending on institutional protocols and clinical recovery.

Overall methodological quality varied across studies (*Appendix S4*). Most trials demonstrated low risk of bias for random sequence generation and allocation concealment, whereas blinding of participants and personnel was frequently rated as high risk due to the nature of the interventions. Blinding of outcome assessment and incomplete outcome data were generally adequate. Selective reporting was mostly judged as low or unclear risk. Importantly, the risk-of-bias patterns did not materially influence the direction of the clinical outcomes observed in this review.

### Pain control and opioid-sparing effects

**Fig. 2:**
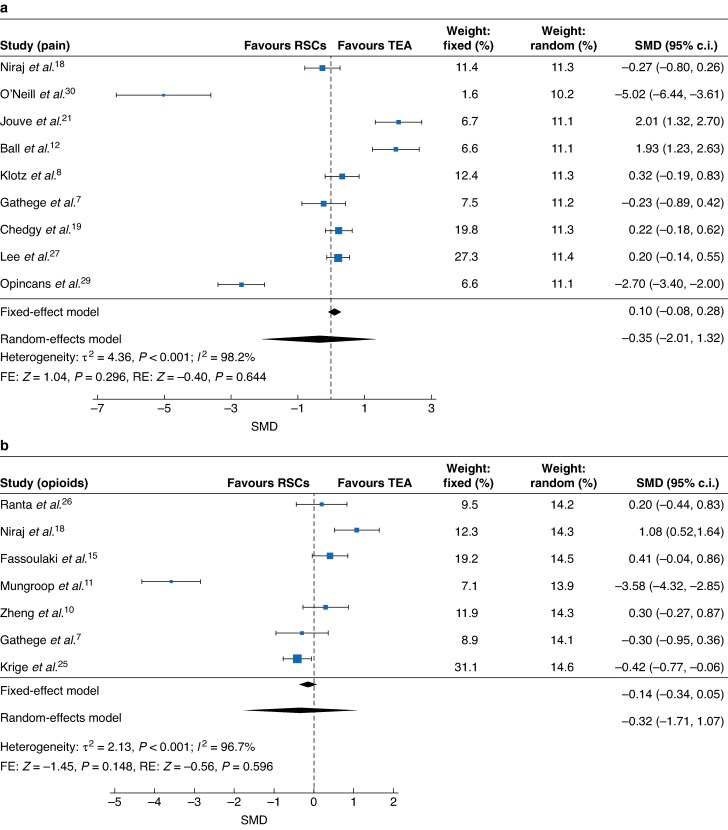
Forest plots of pain scores and opioid consumption in RSC and TEA groups **a** Forest plot of pain score within 24−48 h after surgery. **b** Forest plot of cumulative opioid consumption during hospitalization. Squares denote the study-specific outcome estimates, and the size of each square represents the study-specific weight. Horizontal lines and numbers in parentheses represent the 95% confidence intervals. Diamonds indicate the pooled effect sizes with the corresponding 95% confidence intervals. RSC, rectus sheath catheter; TEA, thoracic epidural analgesia; SMD, standardized mean difference; FE, fixed-effect model; RE, random-effects model.

Across most studies, postoperative pain scores were comparable between RSCs and TEA^6–18^ (*Appendix S5*). Several randomized trials showed slightly lower pain scores with TEA on the day of surgery or during the immediate recovery phase^19–24^; however, this difference typically disappeared by postoperative day 1^19^. This early advantage likely reflects that epidural infusions were initiated intraoperatively, while RSC wound infusions usually began only after skin closure. In the meta-analysis of randomized trials, the pooled SMD showed no significant difference in postoperative pain between RSCs and TEA (random-effects SMD −0.35 (95% c.i. −2.01 to 1.32); *Z* = −0.48; *P* = 0.644), indicating equivalent overall pain relief despite high statistical heterogeneity (*Fig. 2a*). Opioid consumption was similar across most studies, indicating effective pain control with both techniques^13,16,19,25,26^, but some studies showed a trend toward higher opioid consumption in the RSC group during the early postoperative interval^14,15,21^. This was not confirmed in the meta-analysis (random-effects SMD −0.32 (95% c.i. −1.71 to 1.07); *Z* = −0.56; *P* = 0.596) (*Fig. 2b*).

### Adverse events and complications

**Fig. 3:**
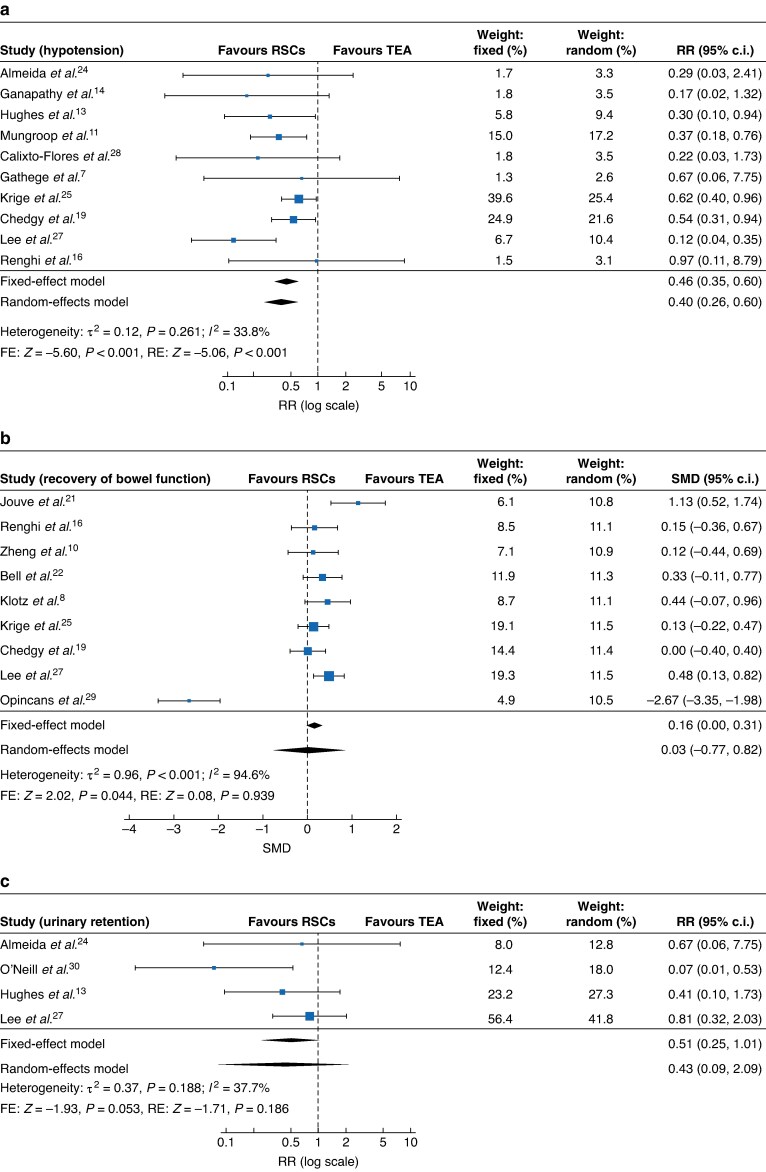
Forest plots of hypotension, bowel function, and urinary retention in RSC and TEA groups **a** Forest plot of hypotension after surgery. **b** Forest plot of length until recovery of bowel function. **c** Forest plot of postoperative urinary retention. Squares denote the study-specific outcome estimates, and the size of each square represents the study-specific weight. Horizontal lines and numbers in parentheses represent the 95% confidence intervals. Diamonds indicate the pooled effect sizes with the corresponding 95% confidence intervals. RSC, rectus sheath catheter; TEA, thoracic epidural analgesia; SMD, standardized mean difference; RR, risk ratio; FE, fixed-effect model; RE, random-effects model.

Hypotension and vasopressor requirement were consistently less frequent with RSCs compared with TEA, reflecting the absence of sympathetic blockade, and resulting in greater haemodynamic stability, particularly among elderly patients^10,11,13,14,19,25,27,28^. This finding was confirmed in the meta-analysis, where RSCs significantly reduced the risk of hypotension (fixed-effect RR 0.46 (95% c.i. 0.35 to 0.60); *Z* = −5.60; *P* < 0.001), with similar results in the random-effects model (RR 0.40 (95% c.i. 0.26 to 0.60); *Z* = −5.06; *P* < 0.001) (*Fig. 3a*). Individual studies showed largely comparable recovery of bowel function in the qualitative analysis^19,20,25,29,30^ (*Appendix S5*). The meta-analysis, which used time in hours until first passage of stool or gas, showed no significant difference between techniques (SMD 0.03 (95% c.i. −0.77 to 0.82); *Z* = 0.08; *P* = 0.939) (*Fig. 3b*).

Regarding urinary function, the duration of indwelling catheters was generally comparable between groups, although several studies reported longer catheterization times with TEA and a higher incidence of urinary retention requiring catheter reinsertion^20,28,30–33^. The meta-analysis showed no significant higher incidence of urinary retention with TEA (RR 0.43 (95% c.i. 0.09 to 2.09); *P* = 0.186) (*Fig. 3c*). Local catheter-site complications (for example displacement and infections) of RSCs and TEA were rare and comparable between both groups^7,8,25,31^.

### Recovery and length of stay

**Fig. 4:**
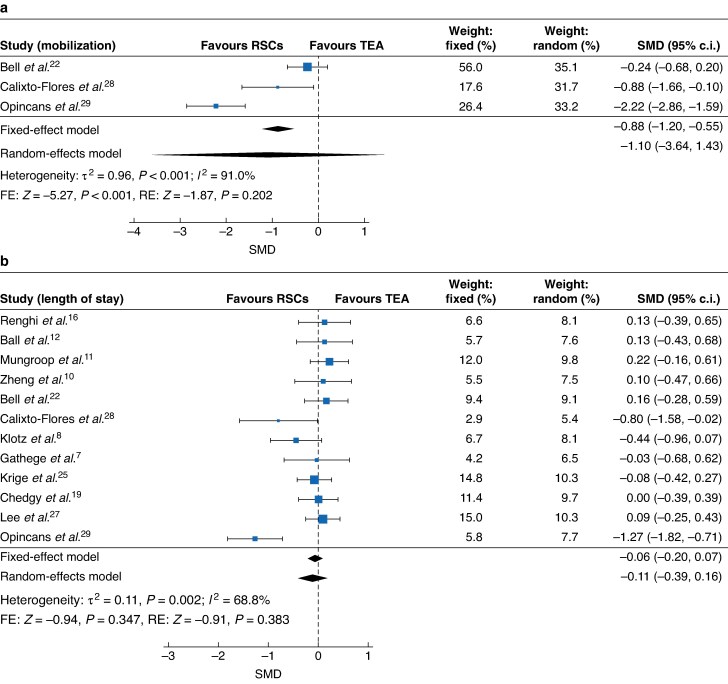
Forest plots of mobilization and length of stay in RSC and TEA groups **a** Forest plot of mobilization after surgery. **b** Forest plot of length of hospital stay. Squares denote the study-specific outcome estimates, and the size of each square represents the study-specific weight. Horizontal lines and numbers in parentheses represent the 95% confidence intervals. Diamonds indicate the pooled effect sizes with the corresponding 95% confidence intervals. RSC, rectus sheath catheter; TEA, thoracic epidural analgesia; SMD, standardized mean difference; FE, fixed-effect model; RE, random-effects model.

Most studies reported similar overall recovery times, while several reported earlier mobilization among those treated with RSCs^28,29,32^. In the fixed-effect meta-analysis of mobilization, RSCs were associated with significantly faster postoperative mobilization (SMD −0.88 (95% c.i. −1.20 to −0.55), *Z* = −5.27; *P* < 0.001), whereas the random-effects model did not reach statistical significance (SMD −1.10 (95% c.i. −3.64 to 1.43); *P* = 0.202) (*Fig. 4a*).

Regarding length of hospital stay, most studies reported a similar number of days of hospitalization, whereas eight studies reported a shorter length of stay in the RSC group, particularly when RSCs were utilized within multimodal analgesia regimens^13,17,23,28–31,33^. However, the meta-analysis showed no difference in length of stay between RSCs and TEA (random-effects SMD −0.11 (95% c.i. −0.39 to 0.16); *P* = 0.383) (*Fig. 4b*). Notably, four of eight studies favouring RSCs were non-randomized cohorts, and were therefore not included in the pooled quantitative analysis.

### Patient satisfaction and acceptability

Overall patient satisfaction was comparable between RSCs and TEA, reflecting generally similar levels of postoperative pain control^10,11^. Even when minor differences in pain intensity were reported, they did not translate into meaningful differences in satisfaction^19,25^. The determinants of satisfaction varied between techniques: TEA patients frequently mentioned anticipatory fear, discomfort during insertion, and fear of limited mobility, whereas RSC patients valued greater independence and fewer side effects, though some expressed uncertainty about pump management or analgesic reliability^34^.

### Costs

Only five studies evaluated economic outcomes; four reported lower overall costs with RSCs^23,25,33,35^, while one found no difference^6^. Reported cost savings ranged from approximately $500 to $6632 per case, mainly due to shorter hospital stay, reduced nursing workload, and lower monitoring requirements compared with TEA.

### Outcomes across surgical subgroups

The predominant discipline across included studies was visceral surgery (12 of 20 (60%)), and the most frequently used incision type was midline laparotomy (12 of 20 (60%)). Studies from non-visceral disciplines such as gynaecology, urology, and vascular surgery reported heterogeneous and largely non-overlapping outcomes, limiting outcome-specific comparisons within these specialties. Therefore, subgroup analyses were predefined according to surgical category (visceral *versus* non-visceral) and incision type (midline laparotomy *versus* other incision types). Analysis revealed several subgroup-specific signals that were not apparent in the overall analyses, largely driven by differences in heterogeneity and the resulting choice of model (*Appendices S6*, *S7*).

For hypotension, the protective effect of RSCs was consistent across strata. In visceral surgery, RSCs reduced hypotension (random-effects RR 0.34 (95% c.i. 0.19 to 0.61); *P* = 0.004), and a similar reduction was observed in non-visceral surgery (fixed-effect RR 0.53 (95% c.i. 0.32 to 0.89); *P* = 0.016). Likewise, the effect remained significant for laparotomy (fixed-effect RR 0.49 (95% c.i. 0.36 to 0.67); *P* < 0.001) and other incisions (fixed-effect RR 0.36 (95% c.i. 0.21 to 0.63); *P* < 0.001).

For urinary retention, effects differed by subgroup. In visceral surgery, no significant difference was found (fixed-effect RR 0.66 (95% c.i. 0.32 to 1.39); *P* = 0.277), whereas, in non-visceral surgery, urinary retention was lower with RSCs (RR 0.07 (95% c.i. 0.01 to 0.53); *P* = 0.009; single-study estimate). Urinary retention did not differ after laparotomy (fixed-effect RR 0.79 (95% c.i. 0.33 to 1.76); *P* = 0.590), but was significantly reduced with RSCs in non-laparotomy incisions (fixed-effect RR 0.23 (95% c.i. 0.07 to 0.72); *P* = 0.012).

For mobilization, the overall analysis was limited by substantial heterogeneity, but subgrouping yielded lower within-stratum heterogeneity and corresponding fixed-effect estimates that reached statistical significance in some strata. In visceral surgery, RSCs were associated with earlier mobilization (SMD −2.22 (95% c.i. −2.86 to −1.59); *P* < 0.001; single-study estimate). In non-visceral surgery, mobilization also favoured RSCs (fixed-effect SMD −0.39 (95% c.i. −0.77 to −0.01); *P* = 0.042), aligning with the observation that heterogeneity was lower within these subgroups compared with the overall pooled analysis. By incision type, mobilization showed no significant difference after laparotomy (random-effects SMD −1.22 (95% c.i. −13.81 to 11.37); *P* = 0.435), but favoured RSCs in non-laparotomy incisions (SMD −0.88 (95% c.i. −1.66 to −0.10); *P* = 0.026; single-study estimate). Across the remaining outcomes (pain, opioids, bowel function, and length of stay), subgroup estimates generally showed no clear or consistent effect modification.

### Sensitivity analyses and exploratory meta-regression

Sensitivity analyses using leave-one-out methods and influence diagnostics were performed for all outcomes (*Appendix S8*). Overall, the pooled estimates remained stable across most analyses, with no single study materially altering the direction or significance of the results. An exception was observed for bowel function, where exclusion of one study resulted in a statistically significant pooled effect, indicating some instability of this estimate.

Exploratory meta-regression analyses were conducted for selected outcomes with sufficient numbers of included studies (pain, opioid consumption, hypotension, bowel function, and length of stay) to assess the potential influence of surgical category, incision type, and publication year (*Appendix S9*). No statistically significant moderators were identified in any of these analyses.

## Discussion

This systematic review and meta-analysis demonstrates that both TEA and RSCs provide effective postoperative analgesia after open surgical procedures, though with distinctly different risk–benefit profiles.

The meta-analysis found comparable outcomes of RSCs and TEA in postoperative pain control and opioid consumption. Although some individual studies showed marginally superior analgesia with TEA during the immediate postoperative interval, these differences typically disappeared by postoperative day 1 and did not translate into improved patient satisfaction. This suggests that factors beyond absolute pain scores, including autonomy, perceived safety, and recovery trajectory, significantly influence the patient experience^34^. However, the absence of statistically significant differences should not be interpreted as absolute absence of differences. The wide confidence intervals observed for pain (SMD −0.35 (95% c.i. −2.01 to 1.32)) and opioid consumption (SMD −0.32 (95% c.i. −1.71 to 1.07)) reflect uncertainty rather than established equivalence. To aid clinical interpretation, the observed SMDs can be translated to the original pain scale. Based on the included studies, a typical standard deviation of approximately 2–3 points on a 0–10 pain scale was observed. Accordingly, an SMD in the observed range would correspond to an absolute difference of roughly 0.5–1.5 points. This suggests that, even if present, the magnitude of the effect is likely to be small. The limited number of studies contributing to several outcomes and the marked heterogeneity may have resulted in underpowered analyses, increasing the risk of type II error. Thus, absence of evidence is not evidence of absence; demonstrating true equivalence would require adequately powered non-inferiority trials.

However, the results suggest a potential advantage of RSCs owing to their superior safety profile. The analysis revealed a reduction in hypotension risk with RSCs compared with TEA, which could be particularly relevant for elderly patients and those with cardiovascular co-morbidities, but also questions the need for intensive haemodynamic monitoring, simplifying postoperative care and potentially improving resource utilization. While functional recovery outcomes showed comparable results with no difference in recovery of bowel function, there was a trend toward reduced urinary retention with RSCs. The ability to avoid prolonged urinary catheter dwell time or reinsertion represents benefits with respect to both patient comfort and prevention of infection.

Subgroup analyses provided additional clinically relevant insights. While the overall analyses were frequently limited by substantial heterogeneity, stratification by surgical category and incision type reduced within-group variability for selected outcomes. Mobilization emerged as significantly faster with RSCs in non-visceral surgery and non-laparotomy incisions, where fixed-effect models could be applied due to lower heterogeneity. Similarly, urinary retention was significantly less frequent with RSCs in non-visceral procedures, predominantly gynaecological and urological surgery, and in non-laparotomy incisions. These findings suggest that the benefits of RSCs may be most pronounced in surgical settings characterized by limited sympathetic stress. Visceral surgical trauma is associated with a pronounced sympathetic-mediated neuroendocrine and metabolic stress response^36^, which may be less effectively attenuated by peripheral or wound-based analgesia techniques. Furthermore, patient selection may influence the detectability of between-group differences; non-visceral surgical patients, particularly urological and gynaecological patients, may differ (for example, on average, be older with more co-morbidities) compared with patients undergoing major visceral procedures. This may result in higher baseline event rates for outcomes such as urinary retention and hypotension, thereby increasing the statistical power needed to detect true differences where they exist. Importantly, these subgroup findings should be interpreted cautiously given the limited number of studies contributing to individual strata, but they nonetheless indicate potential effect modification that warrants further targeted investigation.

The economic implications of the findings are substantial. Among studies reporting cost outcomes, RSCs demonstrated savings ranging from $500 to $6632 per case, primarily driven by reduced length of stay and decreased healthcare professional workload. While the meta-analysis did not show a statistically significant reduction in hospital stay (SMD −0.11, *P* = 0.383), even modest reductions in hospitalization can yield meaningful economic benefits given daily inpatient costs exceeding $1000–3000 in many healthcare systems. Unlike TEA, RSCs allow for potential discharge with catheters in place. This flexibility aligns with the growing emphasis on ambulatory surgery pathways and early hospital discharge, positioning RSCs as a more adaptable technique for modern perioperative care.

Several limitations merit consideration when interpreting the findings. Substantial heterogeneity existed across studies in anaesthetic concentrations (bupivacaine 0.125–0.25% and ropivacaine 0.1–0.375%), infusion rates (2–12 ml/h), and catheter placement and management protocols. An important limitation is that several outcomes may have been underpowered to detect clinically meaningful differences, particularly for urinary retention, mobilization, and patient satisfaction, which were assessed in relatively few studies with modest sample sizes. In addition, variability in outcome definitions and reporting across studies further limited comparability and synthesis. Future studies would benefit from more standardized and consistently reported outcome measures. The wide confidence intervals for most outcomes preclude definitive conclusions regarding equivalence, and the null findings should be interpreted as inconclusive rather than as evidence of no difference. Future studies should prioritize standardized placement protocols to improve outcome consistency and enable better cross-study comparisons as the described techniques varied from subfascial to suprafascial positioning. Further research should determine whether early catheter insertion at the time of fascial incision, with initial bolus administration, or deeper placement (subfascial or preperitoneal) provides superior analgesia through earlier or more targeted local anaesthetic delivery to relevant nerve branches^37^. The inability to blind participants and personnel in most studies introduced potential performance bias, though this reflects the pragmatic reality of comparing these techniques. Additionally, the conversion of non-parametric data to means and standard deviations may have introduced variability in effect estimates. Despite these limitations, the consistency of findings across diverse surgical specialties, including visceral, urological, gynaecological, and vascular surgery, strengthens the generalizability of the conclusions. This study represents the first comprehensive meta-analysis integrating such a broad range of surgical indications, and the emergence of significant effects despite methodological heterogeneity underscores the robustness of the observed patterns. Furthermore, the generalizability of TEA outcomes may vary considerably across clinical settings due to operator-dependent factors, as epidural catheter insertion is a technically demanding procedure with a well-documented learning curve^38^. This represents an inherent limitation of TEA that does not similarly affect RSCs, which require a less specialized insertion technique with a shorter learning curve.

In conclusion, RSCs offer a compelling alternative to TEA for postoperative analgesia in open surgery. While providing comparable pain control, RSCs demonstrate a superior safety profile with reduced risk of hypotension, simplified postoperative management, and potential economic advantages. However, claims of equivalence for outcomes such as pain control, opioid consumption, and length of stay should be interpreted with caution given the limited statistical power and substantial heterogeneity observed. Adequately powered non-inferiority trials are needed to definitively establish whether RSCs provide equivalent analgesia to TEA. As perioperative care continues to evolve toward enhanced recovery protocols and value-based healthcare models, RSC-based multimodal analgesia strategies are well positioned to become standard practice. Future research should focus on optimizing catheter placement techniques, standardizing infusion protocols, and identifying patients who may derive particular benefit from this approach.

## Supplementary Material

znag058_Supplementary_Data

## Data Availability

Additional data can be made available upon reasonable request.
