# Supplementary material for: Rectus sheath catheters versus thoracic epidural analgesia for pain management after open surgery: systematic review and meta-analysis
Source: Br J Surg. 2026 May 13;113(6):znag058. doi: 10.1093/bjs/znag058 (PMC13262230; doi:10.1093/bjs/znag058)
Supplement: znag058_Supplementary_Data [file znag058_supplementary_data.zip › Supplementary_material.docx]

**Rectus sheath catheters versus thoracic epidural analgesia for pain management after open surgery – A systematic review and meta-analysis**

# Christian D. Fankhauser^1,2,3,4^, Stefan Breitenstein^4,5^, Hans Gelpke^5^, Caveh Madjdpour^4,6^, Gesine Meili^7^, Leila Sultan-Beyer^7^, Thomas R. Wyss^8,9^, Ernest Kaufmann^2,3^

^1^ Division of Urology, Department of Surgery, Cantonal Hospital of Winterthur, Winterthur, Switzerland

^2^ Faculty of Health Sciences and Medicine, University of Lucerne, Switzerland

^3^ Clinic for Urology, University Teaching and Research Hospital of the University of Lucerne, Switzerland

^4^ University of Zurich, Zurich, Switzerland

^5^ Department of Surgery, Cantonal Hospital of Winterthur, Winterthur, Switzerland

^6^ Department of Anesthesiology, Cantonal Hospital of Winterthur, Winterthur, Switzerland

^7^ Department of Gynecology and Obstetrics, Cantonal Hospital of Winterthur, Winterthur, Switzerland

^8^ Department of Vascular Surgery, Inselspital, Bern University Hospital, University of Bern, Bern, Switzerland

^9^ Department of Interventional Radiology and Vascular Surgery, Cantonal Hospital of Winterthur, Winterthur, Switzerland

Corresponding Author: Christian D. Fankhauser, Division of Urology, Department of Surgery, Cantonal Hospital of Winterthur, Winterthur, Switzerland, Tel: +41 1 205 21 21, Email: [cdfankhauser@gmail.com](mailto:cdfankhauser@gmail.com), ORCID: 0000-0002-4073-5488

**Supplementary Materials - Index**

| **Supplementary Methods** |  |
| --- | --- |
| **Appendix 1** - Study eligibility, definitions, statistical analysis | *Page 2* |
| **Appendix 2** - Search Strategy | *Page 3* |
| **Supplementary Results** |  |
| **Appendix 3** - Data supplement of included studies | *Page 4* |
| **Appendix 4** - Risk of bias summary | *Page 9* |
| **Appendix 5** - Comparative outcomes of rectus sheath catheters versus thoracic epidural analgesia across included studies | *Page 10* |
| **Appendix 6** - Subgroup analysis for different surgical categories and incision types | *Page 11* |
| **Appendix 7** - Forest plot of subgroup analysis for outcomes in rectus sheath catheter and thoracic epidural anaesthesia groups | *Page 12* |
| **Appendix 8** - Leave-one-out sensitivity analysis for different outcomes | *Page 15* |
| **Appendix 9** - **Exploratory meta-regression analyses** | *Page 16* |
| **References** | *Page 17* |

**Supplementary Methods – Appendix 1** - Study eligibility, definitions, statistical analysis

Study eligibility

Studies were included if they (1) Compared rectus sheath catheter analgesia with thoracic epidural analgesia; (2) Involved open abdominal, pelvic, thoracic, or vascular surgery in adult patients; (3) Reported at least one relevant postoperative outcome (pain, opioid use, complications, recovery, patient satisfaction, or costs).

Outcome selection and definitions

All outcomes reported in each study were systematically screened, and for each outcome domain the measure with the highest cross-study consistency and analyzability was selected. Pain scores were extracted as postoperative pain measured with the visual analogue scale (VAS) or numeric rating scale (NRS) within the first 24 hours (or 48 hours if 24-hour data were unavailable), using mean (standard deviation) when reported or median (interquartile range or range) otherwise. Opioid consumption was recorded as cumulative morphine-equivalent doses. Bowel function was defined as time after surgery in hours until first stool or if not reported, time until first passage of gas. Mobility was defined as time in hours to first out-of-bed activity. Clinically relevant hypotension (needing fluid boluses or vasopressor administration or reduction of TEA) and urinary retention requiring catheter reinsertion were extracted as the number of affected patients, and length of stay as time to hospital discharge in hours. Local catheter complications, patient satisfaction, and cost outcomes could not be subjected to formal meta-analysis because the underlying data were reported using heterogeneous scales, definitions, and cost structures that were not comparable across studies; these outcomes were therefore synthesized narratively.

Statistical analysis

Fixed-effect models assume a common underlying treatment effect and weighted studies by the inverse of their within-study variance. Random-effects models were applied to account for between-study heterogeneity and were estimated using restricted maximum likelihood (REML). Statistical heterogeneity was assessed using Cochran’s Q test, the between-study variance (τ²), and the I² statistic. Pooled estimates were displayed as forest plots with diamonds indicating pooled effects and corresponding 95% confidence intervals. Overall statistical significance of pooled estimates was evaluated using Z tests. When continuous values were reported as medians with interquartile ranges or ranges, they were converted into approximate means and standard deviations using established estimation methods by Wan et al. ^1^ and Luo et al. ^2^, which provide validated formulas for deriving mean and variance estimates from nonparametric summary statistics to enable inclusion in quantitative meta-analysis.

Terminology
In this review, the term rectus sheath catheter (RSC) was used broadly to include various thin, (multi-holed) local wound or fascial plane catheters placed for continuous local anaesthetic infusion; in some studies, epidural catheters were also used for this purpose.

**Supplementary Methods – Appendix 2** - Study eligibility, definitions, statistical analysis

**PICO**

| **Population** | Patients undergoing open surgery |
| --- | --- |
| **Intervention** | Rectus sheath catheter analgesia |
| **Comparison** | Thoracic epidural analgesia |
| **Outcome** | Postoperative outcomes (postoperative pain, complications, recovery) |

**Pubmed
Additional filters:** English, from 1^st^ of January 1990 – until 3^rd^ of November 2025

(("rectus sheath"[tiab] OR "rectus sheath catheter"[tiab] OR "rectus sheath block"[tiab] OR "truncal wall catheter"[tiab]

OR "wound catheter"[tiab] OR "wound infusion"[tiab] OR "continuous wound infusion"[tiab]

OR "wound infiltration"[tiab] OR "continuous wound infiltration"[tiab]

OR "preperitoneal infusion"[tiab] OR preperitoneal[tiab] OR "incisional analgesia"[tiab]

OR "local infiltration"[tiab] OR "local analgesia"[tiab]

OR "continuous local anesthesia"[tiab] OR "continuous local anaesthesia"[tiab]

OR "patient-controlled local analgesia"[tiab] OR PCLA[tiab]

OR "transversus abdominis plane"[tiab] OR "TAP block"[tiab] OR "subcostal TAP"[tiab]

OR ("Rectus Abdominis"[Mesh] AND "Nerve Block"[Mesh]) OR "Infusions, Local"[Mesh])

AND

(epidural"[tiab] OR "epidural analgesia"[tiab] OR "epidural anesthesia"[tiab] OR "epidural anaesthesia"[tiab]

OR "thoracic epidural"[tiab] OR "epidural morphine"[tiab] OR TEA[tiab]

OR "Analgesia, Epidural"[Mesh] OR "Anesthesia, Epidural"[Mesh])

AND

(open[tiab] OR "open surgery"[tiab] OR laparot*[tiab] OR thoracotomy[tiab] OR sternotomy[tiab]

OR aortic[tiab] OR "abdominal aortic"[tiab]

OR cystectomy[tiab] OR prostatectomy[tiab] OR bladder[tiab] OR urologic*[tiab]

OR colorectal[tiab] OR colectomy[tiab] OR rectal[tiab]

OR hepatic[tiab] OR hepatectomy[tiab] OR "liver resection"[tiab]

OR cesarean[tiab] OR caesarean[tiab] OR "cesarean delivery"[tiab]

OR trauma[tiab] OR traumatic[tiab] OR "trauma surgery"[tiab]

OR "orthopedic surgery"[tiab] OR "open fracture"[tiab] OR "fracture fixation"[tiab])

AND

(randomized[tiab] OR randomised[tiab] OR trial[tiab] OR controlled[tiab]

OR comparative[tiab] OR comparison[tiab] OR versus[tiab] OR vs[tiab]

OR noninferiority[tiab] OR "non-inferiority"[tiab]

OR "Randomized Controlled Trial"[Publication Type] OR "Controlled Clinical Trial"[Publication Type]))

AND humans[MeSH Terms]

NOT

("case reports"[Publication Type] OR review[Publication Type] OR editorial[Publication Type] OR letter[Publication Type]

OR (animals[MeSH Terms] NOT humans[MeSH Terms])

OR child[MeSH Terms] OR infant[MeSH Terms] OR adolescent[MeSH Terms]

OR laparoscop*[tiab])

| Rectus sheath | Thoracic epidural | Surgery | Results |
| --- | --- | --- | --- |
| X |  |  | 9.851 |
|  | X |  | 91.359 |
|  |  | X | 2.233.341 |
| X | X | X | 84 |

**Supplementary Figures, Tables and Results**

**Appendix 3** Data supplement of included studies

| **Author** | **Year** | **Country** | **Discipline** | **Surgery** | **Incision type** | **Analgesia type** | **n size per group** | **Age (years)** | **Intervention protocol** | **Drugs administered** | **Study Design** |
| --- | --- | --- | --- | --- | --- | --- | --- | --- | --- | --- | --- |
| **Opincans et. al** | 2025 | Latvia | Visceral | Open Gastrectomy | Upper midline laparotomy | RSC | 30 | 71 | Bilateral retromuscular catheters | bilateral rectus sheath block at the end of surgery, then ‘Easy Pumps’ with 270 mL, 5 mL/h, 0.125% bupivacaine attached to catheters | RCT |
|  | 2025 |  | Visceral | Open Gastrectomy | Upper middle laparotomy | Epi | 34 | 71 | T9/10 epidural | 0.25% bupivacaine at a rate of 5 mL/h |  |
| **Lee et. al.** | 2024 | South Korea | Visceral | Pancreatoduodenectomy | Midline laparotomy | RSC | 70 | 67 | preperitoneal space | 10 ml of 0.75% ropivacaine as a bolus through the catheter, elastomeric infusion pump 300 ml of 0.5% ropivacaine 2 ml/h | RCT |
|  | 2024 |  | Visceral | Open Pancreatoduodenectomy | middle laparotomy | Epi | 64 | 67 | T8 epidural | 0.2% ropivacaine and 1 μg/ml fentanyl, with 4 ml administered as a loading dose. The basal infusion rate was set at 4 ml/h, the patient bolus was 2 ml |  |
| **Davies et. al.** | 2024 | Australia | Visceral | Pancreatoduodenectomy | Midline laparotomy | RSC | 33 | 70 | placed into the rectus sheath after the closure of peritoneum, rectus sheath, and muscle as a single layer | 10 ml ropivacaine as a bolus through the catheter, then continuous ropivacaine | Retrospective cohort |
|  | 2024 |  | Visceral | Open Pancreatoduodenectomy | middle laparotomy | Epi | 41 | 66 | T7-T10 epidural | different agents: ropivacain/bupivacain and fentanyl/pethidin |  |
| **Boesl et. al.** | 2024 | USA | Visceral/Gynecology | Cytoreductive Surgery + HIPEC | Midline laparotomy | RSC | 17 | 66 | placed in the preperitoneal space under direct visualization by the surgeon | 0.5% bupivacaine at 2 ml/h for 5 days | Retrospective cohort |
|  | 2024 |  | Viszeral/Gynecology | Cytoreductive Surgery + HIPEC | middle laparotomy | Epi | 53 | 54 | epidural space | continuous infusion of 0.125% bupivacaine with fentanyl (2 µg/ml) at a rate of 6 ml/hr |  |
| **Chedgy et. al.** | 2023 | Canada/UK | Urology | Radical cystectomy | Lower midline laparotomy | RSC | 46 | 71 | Bilateral Perifix epidural catheters (BBraun) were positioned anterior to the rectus muscle within the rectus sheath | 20 mL of 0.25% bupivacaine bolus each, continuous infusion of bupivacaine 0.125% at a rate of 5 mL/h | RCT |
|  | 2023 |  | Urology | Radical cystectomy | Lower middle laparotomy | Epi | 51 | 69 | T9-T11 epidural | continuous infusion of bupivacaine 0.125% |  |
| **Krige et. al.** | 2022 | UK | Visceral/Urology | rectal/colonic resection + radical cystectomy | Midline laparotomy | RSC | 66 | 67 | inserted bilaterally under ultrasound guidance after induction of general anaesthesia; the catheters were tunnelled subcutaneously to a level above the costal margin | initial 20ml bolus bupivacain 0.25% each, then RSC bolus (40 ml of 0.2% ropivacaine) thereafter every 4h | RCT |
|  | 2022 | UK | Visceral/Urology | rectal/colonic resection + radical cystectomy | Midline laparotomy | Epi | 65 | 67 | T7-T9 (colon), T9-T11 (cystectomy) epidural | a bolus of 10 mL 0.25% bupivacaine with 100 µg fentanyl, then 0.125% bupivacaine and 2 µg/ml fentanyl was commenced at 10 mL/hour |  |
| **Perrin et. al.** | 2021 | New Zealand/ Australia/UK | Visceral | Pancreatic Resection | Left subcostal or upper transverse | RSC | 25 | 64 | ultrasound guided into posterior rectus sheath, for lateral incisions,between the internal oblique and transversus abdominis muscle | bolu 20-30 mL of 0.2%-0.375% ropivacaine, then 20 mL programed bolus of 0.2% ropivacaine every 4h | Retrospective cohort |
|  | 2021 | New Zealand/Australia/UK | Visceral | Pancreatic Resection | Left subcostal or upper transverse | Epi | 47 | 65 | epidural space | bupivacaine 0.125% with fentanyl 2 mg/mL |  |
| **Miller et. al.** | 2021 | USA | Visceral | Colorectal surgery | not specified | RSC | 52 | 61 | placed using ultrasound guidance into the plane between the internal oblique and transversus abdominis muscles using a subcostal approach | ropivacaine 0.1% at 10–15 ml/hr | Retrospective cohort |
|  | 2021 | USA | Visceral | Colorectal surgery | not specified | Epi | 24 | 60 | epidural space | ropivacaine 0.1% at 10–15 ml/hr |  |
| **Kone et. al.** | 2021 | USA | Visceral | Pancreatic Resection | not specified | RSC | 45 | 64 | tunneled pre-peritoneal 19-gauge perforated catheters by the surgeon at the time of abdominal closure | 0.25% bupivacaine at a continuous rate | Retrospective cohort |
|  | 2021 | USA | Visceral | Pancreatic Resection | not specified | Epi | 9 | 63 | epidural space | not specified |  |
| **Gathege et. al.** | 2021 | Kenya | Viszeral/Gynecology | not specified | (Upper) midline laparotomy | RSC | 19 | 50 | multi-hole PajunkD 19G, 60-cm inert catheter onto the sub-fascial space | 10-ml bolus of 0.125% bupivacaine, then 0.125% bupivacaine at an infusion rate of 4–10 ml/hour titrated to response | RCT |
|  | 2021 | Kenya | Viszeral/Gynecology | not specified | (Upper) midline laparotomy | Epi | 19 | 49 | T9/T10 epidural | 0.125% bupivacaine infusion was initiated at rate of 4–10 ml/hour |  |
| **Klotz et. al.** | 2020 | Germany | Visceral/Vascular | Elective upper abdominal surgery | Midline laparotomy | RSC | 31 | 62 | placed suprafascially in the surgical site of the midline laparotomy at the end of surgery after closure of the abdominal fascia and before closure of the subcutaneous tissue and skin | Ropivacain 0.2% isobar at 5ml/h | RCT |
|  | 2020 | Germany | Visceral/Vascular | Elective upper abdominal surgery | Midline laparotomy | Epi | 31 | 62 | T7-T9 epidural | ropivacain 0.2% with sufentanil at 6-10ml/h administered for 3 days |  |
| **Calixto-Flores et. al.** | 2020 | Colombia | Urology | Donor nephrectomy | not specified | RSC | 15 | 40 | transverse abdominal plane (space located between the internal oblique abdominal muscle and the abdominal transverse muscle | 15 mL of 0.375% ropivacaine bolus, then ropivacaine 0.2% 2ml/h | RCT |
|  | 2020 | Colombia | Urology | Donor nephrectomy | not specified | Epi | 15 | 37 | T12/L1 epidural | 10 mL of ropivacaine 0.375% bolus, then ropivacaine 0.2% 2ml/h |  |
| **Bell et. al.** | 2019 | UK | Visceral | Liver Resection | Mostly Reverse L/Midline | RSC | 42 | 65 | 2 multiperforated Painkwell (PeakMedical) catheters inserted into the transversus abdominis plane laterally and the posterior rectus sheath medially | 20 mL 0.5% bupivacaine bolus, then infusion of 0.25% bupivacaine at 4 mL/h per catheter | RCT |
|  | 2019 | UK | Visceral | Liver Resectin | Mostly Reverse L/Midline | Epi | 41 | 67 | T6-T10 epidural | fentanyl (2 mg/mL) and local anesthetic (0.15% bupivacaine) at 6–10 mL/h |  |
| **Che et. al.** | 2017 | China | Visceral | Partial Hepatectomy | Right subcostal incision | RSC | 10 | 55 | between the transversus abdominus and rectus abdominis under direct vision | elastomeric pump with 300 ml of 0.4% lidocaine continuous for 72h | Prospective comparative |
|  | 2017 | China | Visceral | Partial Hepatectomy | Right subcostal incision | Epi | 22 | 51 | T9/T10 epidural | 0.2% ropivacaine at 4 ml/h with a bolus of 4 ml and a lockout time of 15 minutes |  |
| **Zheng et. al.** | 2016 | China | Visceral | Gastrocetomy | (Upper) midline laparotomy | RSC | 25 | 62 | two multiholed catheters were inserted by the surgeons through a separate puncture of the layer of deep fascia adjacent to the surgical incision before closure | 0.3% ropivacaine 5 mL/h | RCT |
|  | 2016 | China | Visceral | Gastrocetomy | (Upper) midline laparotomy | Epi | 25 | 62 | T7/T8 epidural | 0.1% ropivacaine and 20 µg/mL morphine at 6-8 mL/h |  |
| **Mungroop et. al.** | 2016 | Netherlands | Visceral | hepato-pancreato-biliary surgery | Subcostal right laparotomy/ Midline laparotomy | RSC | 55 | n.a. | wound catheters were placed in the subfascial (ie, pre-peritoneal) space under direct vision | bolus of total 30 mL bupivacaine 0·25%, then pumps with a total of 12 mL/h bupivacaine 0.125% | RCT |
|  | 2016 | Netherlands | Visceral | hepato-pancreato-biliary surgery | Subcostal right laparotomy/ Midline laparotomy | Epi | 47 | n.a. | T7-T10 epidural | bupivacaine 0.125% and sufentanil 1 μg/mL with a fi xed rate of 6 mL/h and bolus of 2 mL and a lockout time of 20 min |  |
| **Ball et. al.** | 2016 | Italy | Vascular | Abdominal Aortic Aneurism repair | Midline laparotomy | RSC | 26 | 75 | two fenestrated catheters were placed by the surgeon between the peritoneum and the fascia | bolus 10 mL 0.5% levobupivacaine, then elastomeric pumps loaded with levobupivacaine 0.25% and a continuous infusion at a fixed rate of 4 mL/h | RCT |
|  | 2016 | Italy | Vascular | Abdominal Aortic Aneurism repair | Midline laparotomy | Epi | 25 | 74 | T7-T9 epidural | levobupivacaine 0.12% plus sufentanil 0.4 µg/ mL at a fixed 5 mL/h |  |
| **Hughes et. al** | 2015 | UK | Visceral | Partial Hepatectomy | Right subcostal incision | RSC | 49 | 63 | ON-Q® dual-limb 12⋅5-cm Painbuster® (B. Braun, Sheffield, UK) inserted. lateral: between transversus abdominis and internal oblique muscles, medial: in the rectus sheath posterior to the muscle | bolus 40ml 0.125% levobupivacaine, then elastomeric reservoir 0.375% levobupivacaine rate of 4ml/h | RCT |
|  | 2015 | UK | Visceral | Partial Hepatectomy | Right subcostal incision | Epi | 44 | 63 | T8/T9 epidural | block with 10ml levobupivacaine with 100μg fentanyl, then 0.1% levobupivacaine with 2 μg/ml fentanyl |  |
| **Ganapathy et. al.** | 2015 | Canada | Visceral | Abdominal Surgery | Midline laparotomy | RSC | 26 | 62 | bilateral LM-TAP catheters inserted using a linear ultrasound probe lateral to the edge of the rectus abdominis muscle, inferior TAP catheter was inserted through the Tuohy needle after distending the transversus plane | elastomeric pump via a Y-connector to deliver ropivacaine 0.35% at a rate of 4 to 5 ml/h for 72 h | RCT |
|  | 2015 | Canada | Visceral | Abdominal Surgery | Midline laparotomy | Epi | 24 | 58 | T7-T9 epidural | bupivacaine 0.1% with hydromorphone 10 mg/ml at a rate of 8 ml/h and continued for 72 h |  |
| **Fassoulaki et. al.** | 2014 | Greece | Gynecology | Hysterectomy/Myomectomy | Pfannestiel | RSC | 40 | 44 | “PAINfusor” multihole catheter along the 75 mm distal part (Plan 1 Health, Amaro, Italy) was inserted above the fascia | 0.375% ropivacaine 2ml/h | RCT |
|  | 2014 | Greece | Gynecology | Hysterectomy/Myomectomy | Pfannestiel | Epi | 40 | 41 | L3/L4 epidural | 10 mL of 0.75% ropivacaine then 10 mL of 0.2% ropivacaine every 6h |  |
| **Renghi et. al.** | 2013 | Italy | Vascular | Abdominal Aortic Surgery | left subcostal | RSC | 30 | 69 | subfascial and subcutaneous placement of a double multiperforated catheter | levobupivacaine, 0.25% at 4 mL/h | RCT |
|  | 2013 | Italy | Vascular | Abdominal Aortic Surgery | left subcostal | Epi | 29 | 72 | T6/T7 epidural | levobupivacaine, 0.25% at 4 mL/h |  |
| **Jouve et. al.** | 2013 | France | Visceral | Colorectal surgery | Midline laparotomy | RSC | 26 | 68 | multi-holed catheter was positioned between the closed parietal peritoneum and the transversalis fascia | 10 ml 0.2% ropivacaine was administered, fol- lowed by a continuous infusion at a constant rate of 10 ml/h | RCT |
|  | 2013 | France | Visceral | Colorectal surgery | Midline laparotomy | Epi | 24 | 63 | T8-T11 epidural | 0.375% ropivacaine continuous infusion at 10 ml/h |  |
| **Gebhardt et. al.** | 2013 | USA | Thoracic/Cardiovascular surgery | Not specified | Thoracotomy | RSC | 26 | 61 | multi-orifice tube that was inserted along the inferior border of the rib along the incision so that the tip lay in a space created underneath the longissimus dorsi muscle, placing the catheter close to the costovertebral joint | bolus 5 mL of ropivacaine 0.5% before skin closure. After skin closure ON-Q pump, containing 0.5% ropivacaine with 2 mL/h | Retrospective cohort |
|  | 2013 | USA | Thoracic/Cardiovascular surgery | Not specified | Thoracotomy | Epi | 24 | 60 | T4-T8 epidural | bupivicaine 0.075% or 0.1%, with hydromorphone [10 mg/mL, 5 mg/mL, or 2 mg/mL] or fentanyl [5 mg/mL] |  |
| **Finch et.al.** | 2013 | UK | Gynecology | Not specified | Midline laparotomy | RSC | 29 | n.a. | between the peritoneum and the rectus muscle | 20 ml 0.25% plain levo-bupivacaine is injected into each catheter, repeated 6-hourly | Retrospective cohort |
|  | 2013 | UK | Gynecology | Not specified | Midline laparotomy | Epi | 16 | n.a. | Not specified | Not specified |  |
| **Tilleul et. al.** | 2012 | France | Visceral | Upper digestive/colorectal/hepato-biliary | not specified | RSC | 20 | 60 | 20 G multiholed (fenestrated) catheter (Painfusor Baxter Healthcare Corp., Maurepas, France positioned in a preperitoneal position | 10 ml bolus of 0.2% ropivacaine, then elastomeric pump 10 ml/h for 48 h | Cost-effectiveness analysis (Prospective cohort) |
|  | 2012 | France | Visceral | Upper digestive/colorectal/hepato-biliary | not specified | Epi | 22 | 53 | T7-T10 epidural | bolus of 4–6 ml of a mixture of 1.25 mg/ml levobupivacaine and 0.5 mg/ml sufentanil followed by a patient-controlled epidural system (4–6 ml/ml continuous infusion rate with 2–4 ml bolus with a 20 min lock-out time) |  |
| **O'Neill et. al.** | 2012 | Portugal | Gynecology | Cesarean Section | Pfannestiel | RSC | 29 | n.a. | multiorifice wound catheter placed below the fascia | 5 mL/h ropivacaine 2 mg/mL | RCT |
|  | 2012 | Portugal | Gynecology | Cesarean Section | Pfannestiel | Epi | 29 | n.a. | epidural space | epidural bolus of morphine 2 mg every 12 hours |  |
| **Niraj et. al.** | 2011 | UK | Visceral/Urology | upper abdominal surgery | extended right subcostal, chevron, transverse | RSC | 27 | 64 | ultrasound guided TAP catheters in the subcostal transversus abdominis plane | 8-hourly bolus injections of 1 mg/kg bupivacaine 0.375% through each TAP catheter | RCT |
|  | 2011 | UK | Visceral | upper abdominal surgery | extended right subcostal, chevron, transverse | Epi | 31 | 64 | T7-T9 epidural | bupivacaine 0.125% with fentanyl 2 ug/ml. started at 6 ml/h and was increased up to 12 ml/h |  |
| **Gross et. al.** | 2011 | USA | Visceral | not specified | Midline laparotomy | RSC | 99 | 57 | within the preperitoneal space, below the right and left rectus fascia | elastomeric pumps 0.5% bupivacaine at a constant rate of 2 mL/h | Retrospective cohort |
|  | 2011 | USA | Visceral | not specified | Midline laparotomy | Epi | 141 | 56 | epidural space | not specified |  |
| **de Almeida et. al.** | 2011 | Brazil | Visceral | not specified | Midline laparotomy | RSC | 19 | 50 | between the recent sutured aponeurosis and the deepest portion of the subcutaneous tissue, along the entire incision | 10 mL bolus of ropivacaine 0.2%, then rate of 5ml/h | RCT |
|  | 2011 | Brazil | Visceral | not specified | Midline laparotomy | Epi | 19 | 56 | epidural space | 0.75% ropivacaine 40 mL, fentanyl 250 µg, and saline 33 mL. Infusion rate 2 mL/h |  |
| **Aung et. al.** | 2010 | New Zealand | Visceral | elective/emergency laparotomies | Midline laparotomy | RSC | 27 | n.a. | into the midline fascia into the space between rectus muscle and posterior rectus sheath before closure of deep fascia | loading dose of 0.75% ropivacaine followed by continuous infusion of 0.2% ropivacaine at 5ml/h | Retrospective cohort |
|  | 2010 | New Zealand | Visceral | elective/emergency laparotomies | Midline laparotomy | Epi | 12 | n.a. | epidural space | not specified |  |
| **Ranta et. al.** | 2006 | Sweden | Gynecology | Cesarean Section | Pfannestiel | RSC | 20 | 29 | along the entire length of the surgical wound under the abdominal fascia | 10mL boluses of 0.25% levobupivacaine | RCT |
|  | 2006 | Sweden | Gynecology | Cesarean Section | Pfannestiel | Epi | 20 | 28 | L1/L2 epidural | 10mL boluses of 0.125% levobupivacaine |  |

**Appendix 4** Risk of bias summary. (A) Domain-level risk of bias summary. This panel summarizes the proportion of studies judged as having low, unclear, or high risk of bias across the Cochrane risk-of-bias domains. (B) Study-level risk of bias assessment. This panel displays the individual risk-of-bias judgement for each included study across all assessed domains, using a traffic-light coding scheme.

**A**


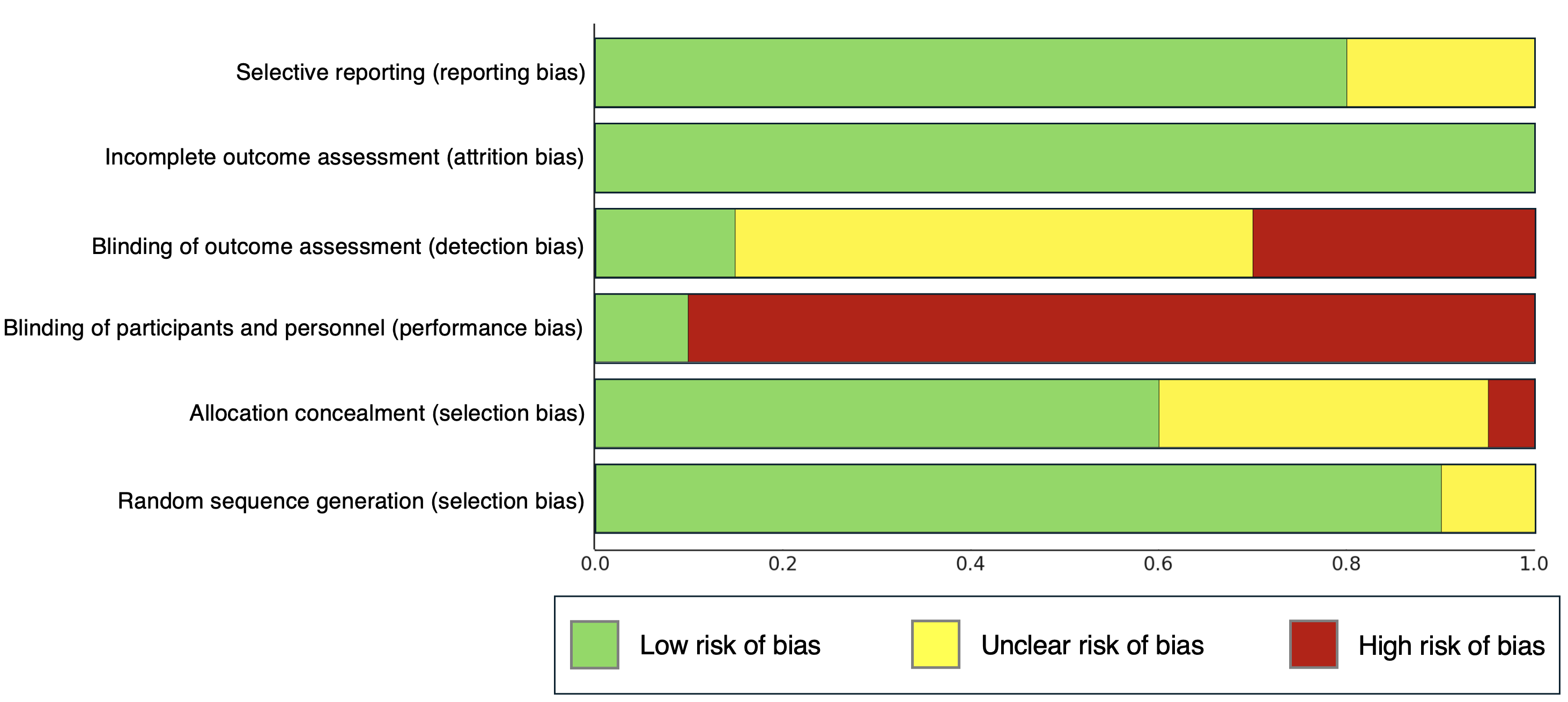


**B**

**
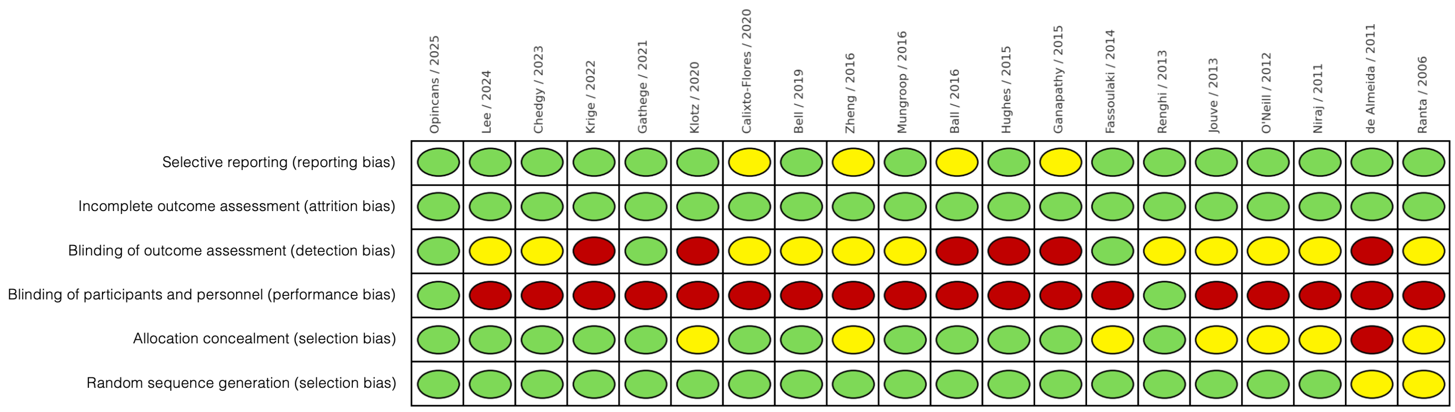
**


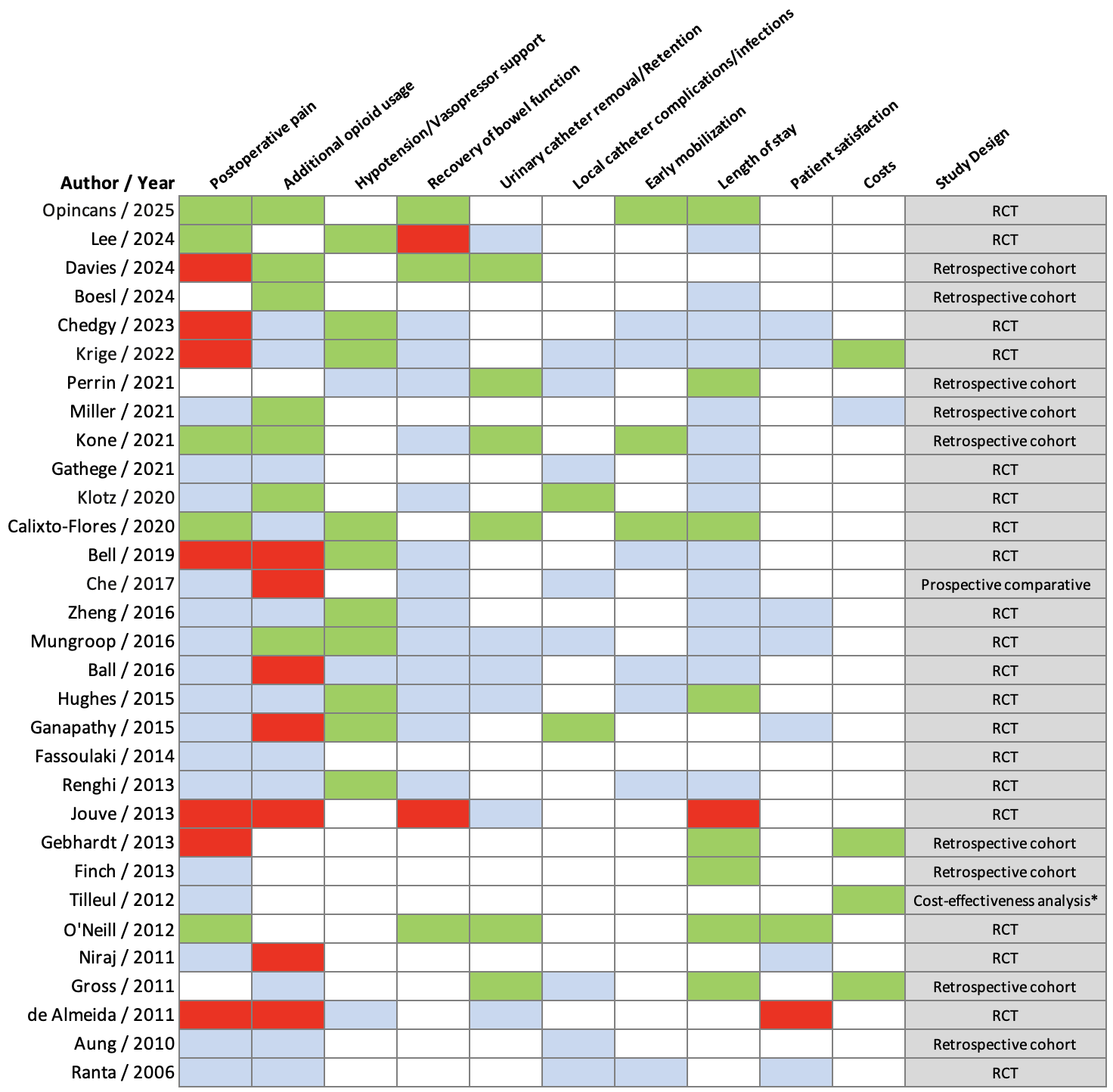
**Appendix 5** Comparative outcomes of rectus sheath catheters versus thoracic epidural analgesia across included studies. Heatmap summarizing the direction of results for assessed outcomes. Each row represents one study. Green cells indicate outcomes favoring rectus sheath catheters, red cells outcomes favoring thoracic epidural analgesia, and blue cells indicate similar results or small non-significant trends between both groups. White cells denote outcomes not assessed in the respective study.
Abbreviations: RCT: Randomized controlled trial

*Cost-effectiveness analysis based on a prospective observational cohort.

**Appendix 6** Subgroup analysis for different surgical categories and incision types

(A) Comparison of different surgical categories, “non-visceral” includes Gynecology, Urology and Vascular Surgery. (B) Comparison of different incision types, “Other Incision” includes Pfannenstiel and left or right subcostal incision. Random-effects model was used when the P value for heterogeneity test was < .05, otherwise the fixed-effect model was used. Abbreviations: SMD: standardized mean difference, RR: Risk Ratio, CI: confidence interval, K number of studies

**A**

| Outcome | Group | K | Test of treatment effect | | | | Model | Test of heterogeneity | | |
| --- | --- | --- | --- | --- | --- | --- | --- | --- | --- | --- |
|  |  |  | SMD | RR | 95% CI | P value |  | τ^2^ | I^2^ | P value |
| Pain | Visceral | 6 | -0.11 |  | -1.69, 1.47 | 0.869 | Random | 2.14 | 96.6% | < 0.001 |
|  | Non-Visceral | 3 | -0.91 |  | -9.86, 8.04 | 0.704 | Random | 12.61 | 98.9% | < 0.001 |
| Opioids | Visceral | 5 | -0.57 |  | -2.77, 1.62 | 0.510 | Random | 3.01 | 97.5% | < 0.001 |
|  | Non-Visceral | 2 | 0.34 |  | -0.03, 0.70 | 0.072 | Fixed | 0 | 0% | 0.594 |
| Hypotension | Visceral | 7 |  | 0.43 | 0.31, 0.60 | **< 0.001** | Fixed | 0.21 | 45.1% | 0.133 |
|  | Non-Visceral | 3 |  | 0.53 | 0.32, 0.89 | **0.016** | Fixed | 0 | 0% | 0.612 |
| Bowel function | Visceral | 6 | -0.05 |  | -1.43, 1.33 | 0.931 | Random | 1.61 | 96.5% | < 0.001 |
|  | Non-Visceral | 3 | 0.15 |  | -0.11, 0.41 | 0.250 | Fixed | 0 | 0% | 0.552 |
| Urinary retention | Visceral | 3 |  | 0.66 | 0.32, 1.39 | 0.277 | Fixed | 0 | 0% | 0.738 |
|  | Non-Visceral | 1 |  | 0.07 | 0.01, 0.53 | **0.009** | - | - | - | - |
| Mobilization | Visceral | 1 | -2.22 |  | -2.86, -1.59 | **< 0.001** | - | - | - | - |
|  | Non-Visceral | 2 | -0.39 |  | -0.77, -0.01 | **0.042** | Fixed | 0.10 | 49.6% | 0.159 |
| Length of stay | Visceral | 7 | -0.18 |  | -0.65, 0.28 | 0.373 | Random | 0.18 | 77.9% | < 0.001 |
|  | Non-Visceral | 5 | 0.02 |  | -0.20, 0.24 | 0.860 | Fixed | 0 | 0% | 0.295 |

**B**

| Outcome | Group | K | Test of treatment effect | | | | Model | Test of heterogeneity | | |
| --- | --- | --- | --- | --- | --- | --- | --- | --- | --- | --- |
|  |  |  | SMD | RR | 95% CI | P value |  | τ^2^ | I^2^ | P value |
| Pain | Laparotomy | 7 | 0.25 |  | -1.19, 1.69 | 0.686 | Random | 2.31 | 97.0% | < 0.001 |
|  | Other Incision | 2 | -2.60 |  | -32.79, 27.59 | 0.472 | Random | 10.99 | 97.4% | < 0.001 |
| Opioids | Laparotomy | 3 | -0.23 |  | -0.50, 0.04 | 0.095 | Fixed | 0.08 | 54.6% | 0.109 |
|  | Other Incision | 4 | -0.46 |  | -3.80, 2.88 | 0.689 | Random | 4.28 | 98.0% | < 0.001 |
| Hypotension | Laparotomy | 6 |  | 0.49 | 0.36, 0.67 | **< 0.001** | Fixed | 0.31 | 59.1% | 0.099 |
|  | Other Incision | 4 |  | 0.36 | 0.21, 0.63 | **< 0.001** | Fixed | 0 | 0% | 0.779 |
| Bowel function | Laparotomy | 8 | 0.09 |  | -0.86, 1.03 | 0.831 | Random | 1.17 | 95.6% | < 0.001 |
|  | Other Incision | 1 | 0.15 |  | -0.36, 0.67 | 0.562 | - | - | - | - |
| Urinary retention | Laparotomy | 2 |  | 0.79 | 0.33, 1,76 | 0.590 | Fixed | 0 | 0% | 0.886 |
|  | Other Incision | 2 |  | 0.23 | 0.07, 0.72 | **0.012** | Fixed | 0.67 | 47.0% | 0.170 |
| Mobilization | Laparotomy | 2 | -1.22 |  | -13.81, 11.37 | 0.435 | Random | 1.89 | 96.1% | < 0.001 |
|  | Other Incision | 1 | -0.88 |  | -1.66, -0.10 | **0.026** | - | - | - | - |
| Length of stay | Laparotomy | 9 | -0.13 |  | -0.47, 0.20 | 0.385 | Random | 0.12 | 68.9% | 0.004 |
|  | Other Incision | 3 | 0.05 |  | -0.23, 0.34 | 0.710 | Fixed | 0.16 | 67.6% | 0.068 |

**Appendix 7** Forest plot of subgroup analysis for outcomes in rectus sheath catheter and thoracic epidural anaesthesia groups.

Forrest plots of different outcomes analysed across 4 separate subgroups: Visceral and non-visceral surgery, laparotomy, and other incision types.

A: Pain score within 24(-48h) after surgery.

B: Cumulative opioid consumption during hospitalization.

C: Hypotension after surgery.

D: Length until recovery of bowel function.

E: Postoperative urinary retention.

F: Mobilization after surgery.

G: Length of hospital stay.

Squares denote the study-specific outcome estimates, and the size of the square represents the study-specific weight. Horizontal lines and numbers in parentheses represent the 95% CI. Diamonds indicate the pooled effect size with the corresponding 95% CI. If the 95% CI was very large expanding the scale, a double arrow was used for clarification. Abbreviations: TEA: thoracic epidural analgesia; RSC: rectus sheath catheters. SMD: standardized mean difference, RR: relative risk CI: confidence interval, W: weight, FE: fixed effect model, RE: random effects model


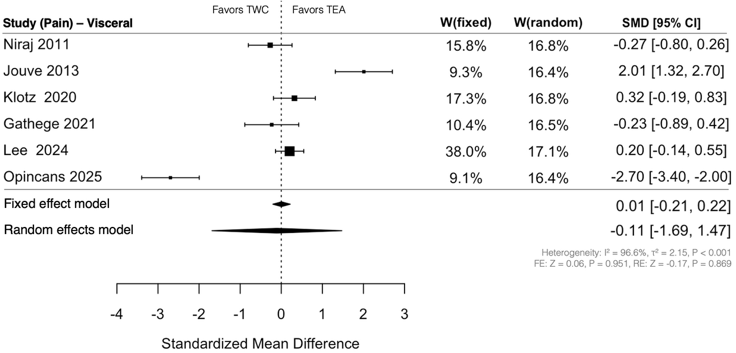

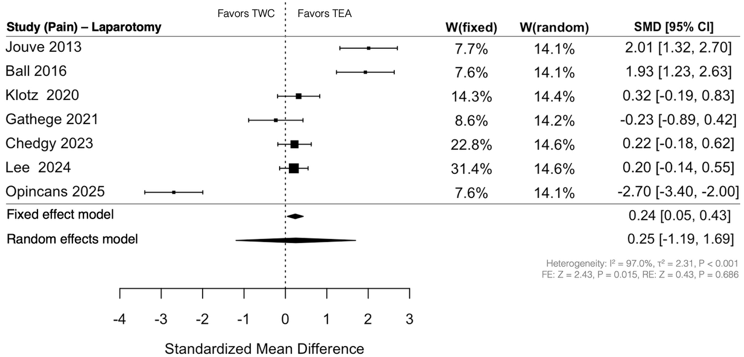


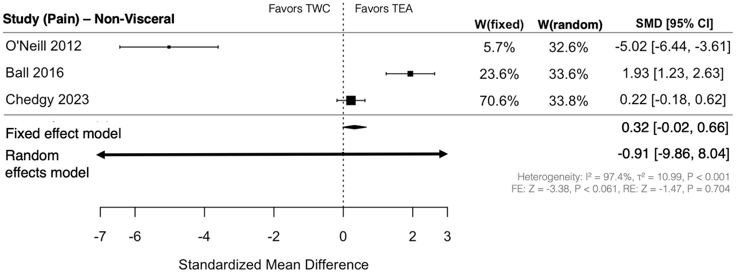

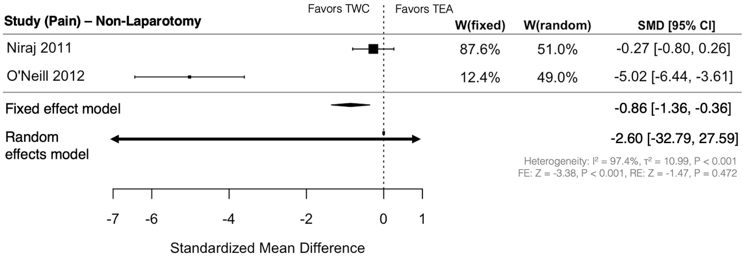


**A**

**A**

**B**


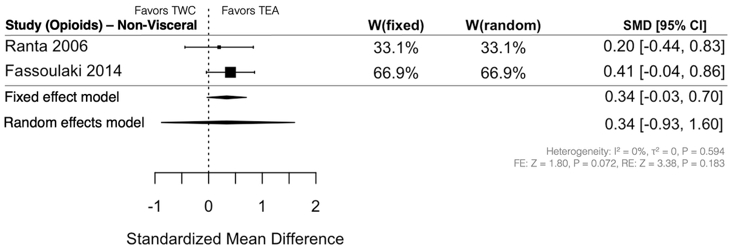

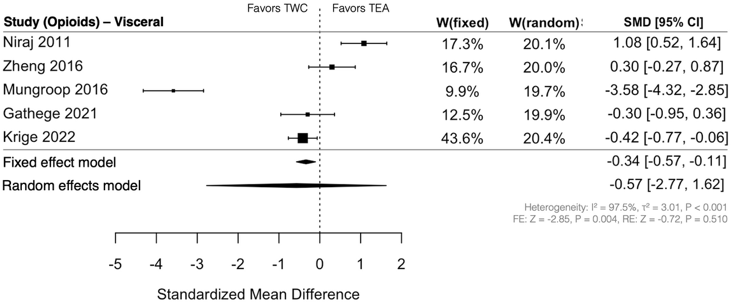

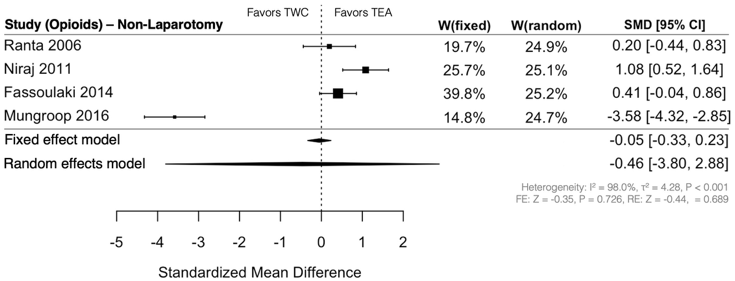

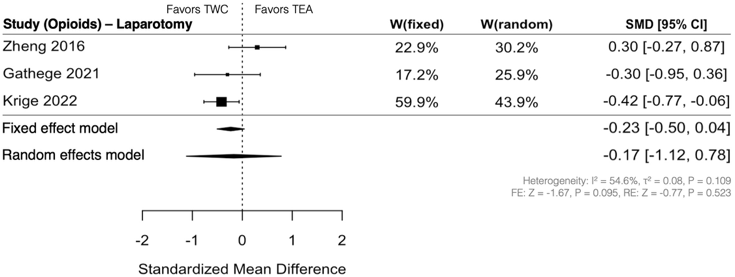


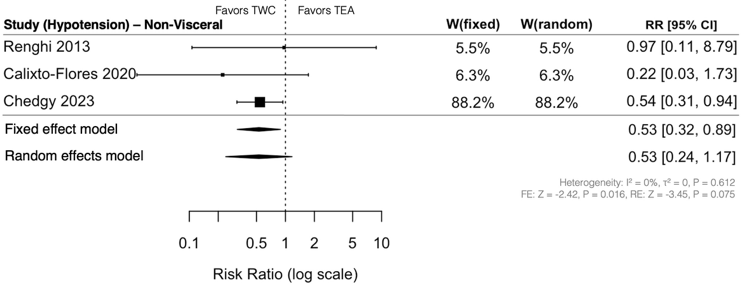

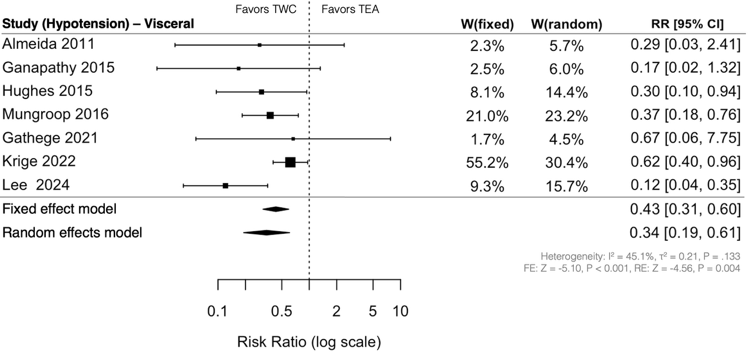

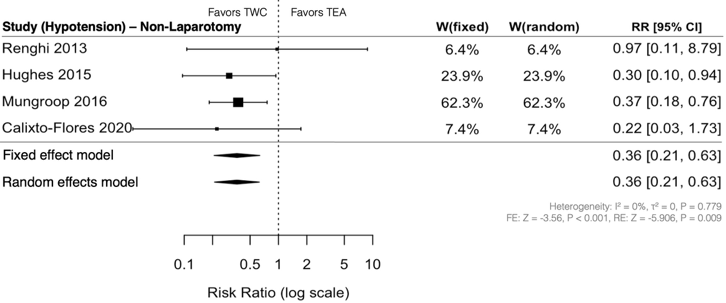

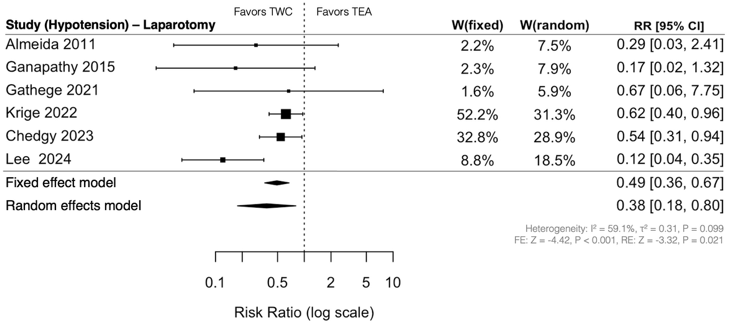


**C**

**D**


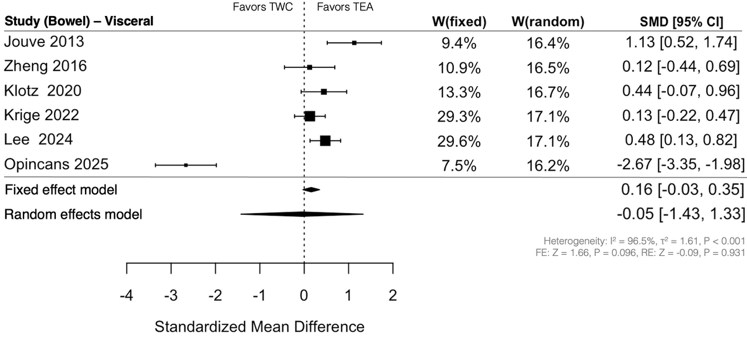

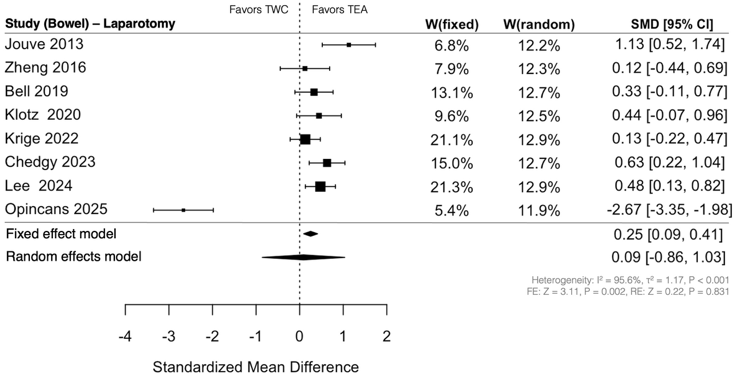


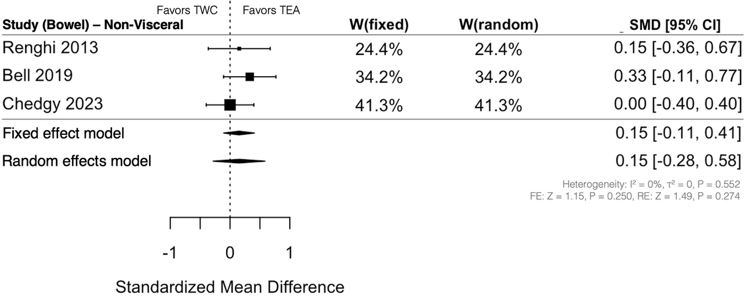

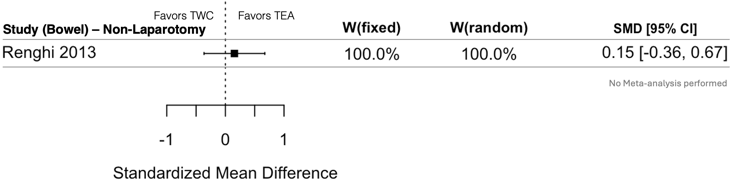


**E**


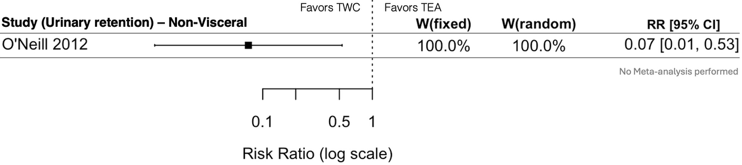

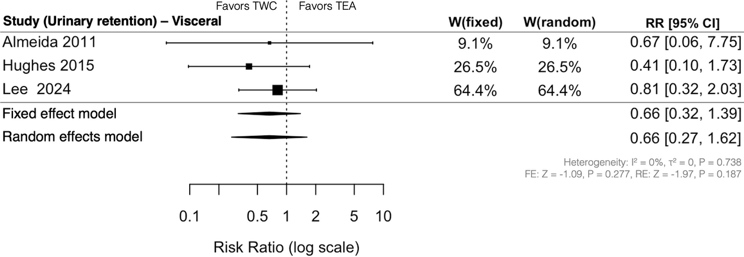

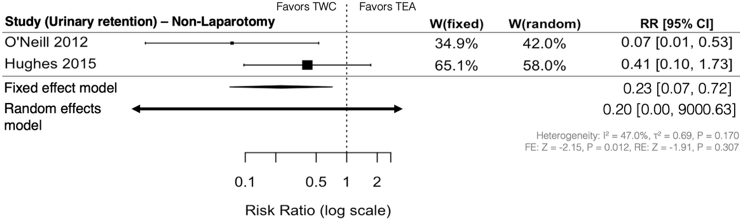

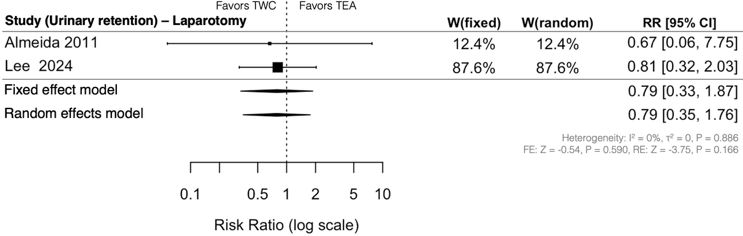


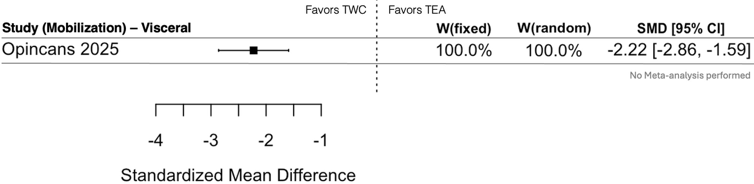

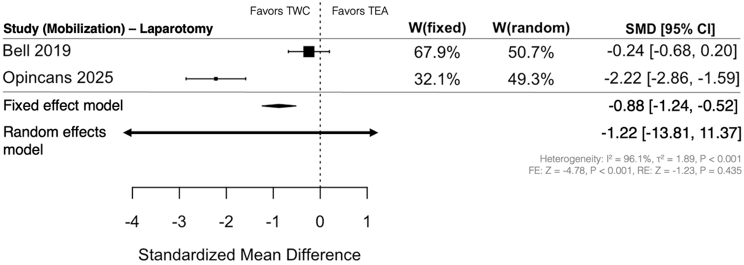


**F**

**
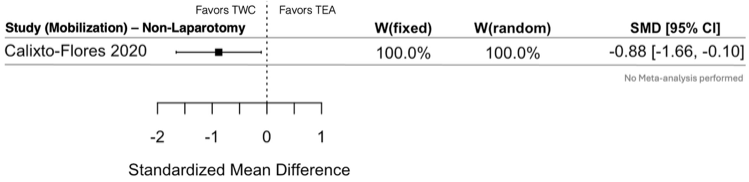
**
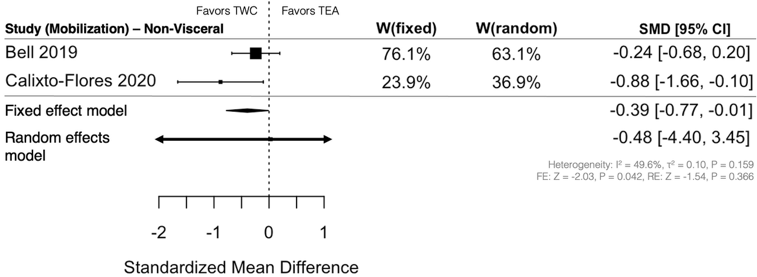


**G**

**
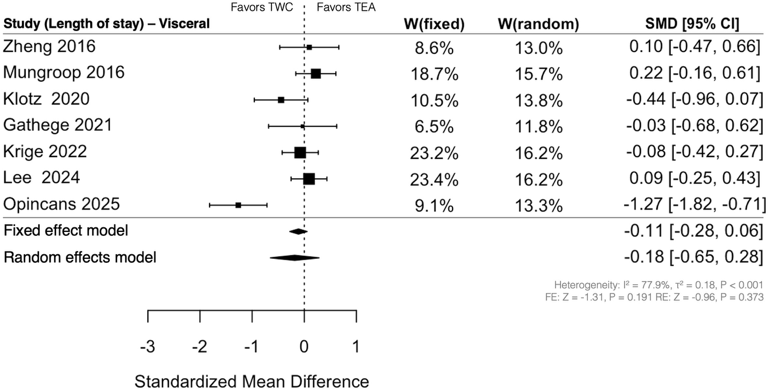

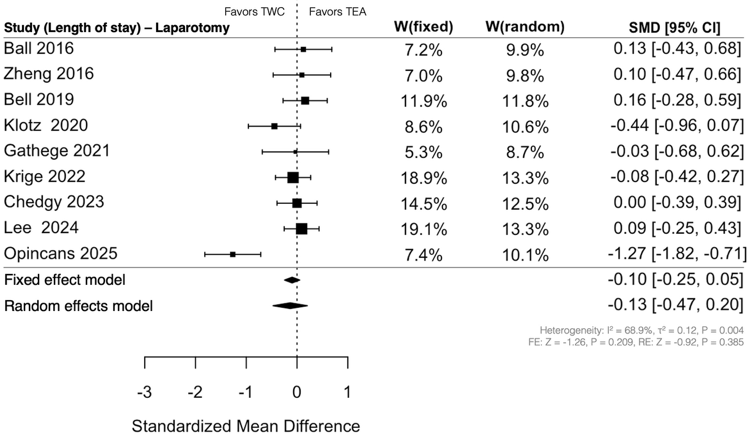
**

**
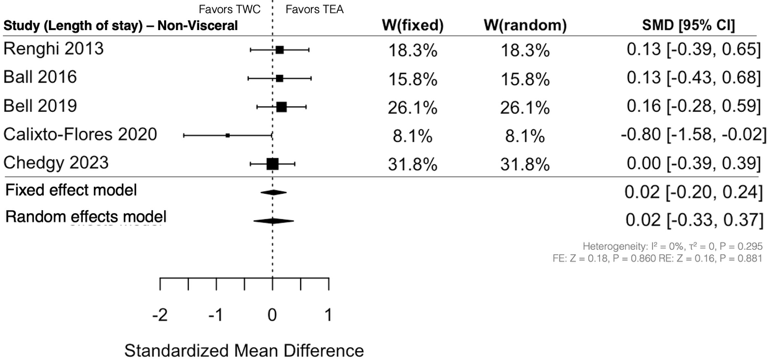

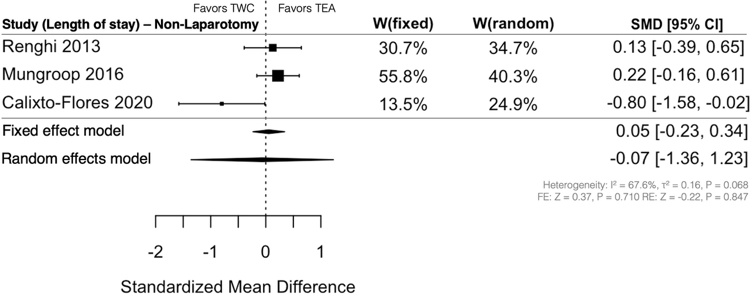
**

**Appendix 8** Leave-one-out sensitivity analysis for different outcomes

Abbreviations: SMD: standardized mean difference, RR: relative risk CI: confidence interval,

| **Pain** | **Opioids** |
| --- | --- |
| \| **Omitted study** \| **SMD** \| **95% CI** \| **P value** \| \| --- \| --- \| --- \| --- \| \| Niraj 2011 \| -0.36 \| -2.31, 1.58 \| 0.672 \| \| O'Neill 2012 \| 0.19 \| -1.03, 1.40 \| 0.729 \| \| Jouve 2013 \| -0.64 \| -2.40, 1.13 \| 0.420 \| \| Ball 2016 \| -0.63 \| -2.41, 1.15 \| 0.429 \| \| Klotz 2020 \| -0.44 \| -2.37, 1.49 \| 0.608 \| \| Gathege 2021 \| -0.37 \| -2.31, 1.57 \| 0.668 \| \| Chedgy 2023 \| -0.43 \| -2.36, 1.51 \| 0.619 \| \| Lee 2024 \| -0.42 \| -2.36, 1.51 \| 0.620 \| \| Opincans 2025 \| -0.05 \| -1.81, 1.71 \| 0.949 \| \|  \|  \|  \|  \| | \| **Omitted study** \| **SMD** \| **95% CI** \| **P value** \| \| --- \| --- \| --- \| --- \| \| Ranta 2006 \| -0.41 \| -2.11, 1.30 \| 0.569 \| \| Niraj 2011 \| -0.55 \| -2.13, 1.02 \| 0.409 \| \| Fassoulaki 2014 \| -0.44 \| -2.13, 1.25 \| 0.531 \| \| Zheng 2016 \| -0.42 \| -2.12, 1.28 \| 0.551 \| \| Mungroop 2016 \| 0.20 \| -0.37, 0.77 \| 0.403 \| \| Gathege 2021 \| -0.32 \| -2.05, 1.40 \| 0.650 \| \| Krige 2022 \| -0.30 \| -2.04, 1.43 \| 0.671 \| |
| **Hypotension** | **Bowel function** |
| \| **Omitted study** \| **RR** \| **95% CI** \| **P value** \| \| --- \| --- \| --- \| --- \| \| Almeida 2011 \| 0.40 \| 0.25, 0.63 \| 0.002 \| \| Renghi 2013 \| 0.38 \| 0.25, 0.59 \| 0.001 \| \| Ganapathy 2015 \| 0.41 \| 0.27, 0.63 \| 0.001 \| \| Hughes 2015 \| 0.40 \| 0.25, 0.65 \| 0.002 \| \| Mungroop 2016 \| 0.39 \| 0.23, 0.64 \| 0.002 \| \| Calixto-Fl. 2020 \| 0.41 \| 0.26, 0.63 \| 0.002 \| \| Gathege 2021 \| 0.39 \| 0.25, 0.61 \| 0.001 \| \| Krige 2022 \| 0.34 \| 0.22, 0.54 \| <0.001 \| \| Chedgy 2023 \| 0.35 \| 0.22, 0.58 \| 0.001 \| \| Lee 2024 \| 0.50 \| 0.39, 0.65 \| <0.001 \| \|  \|  \|  \|  \| | \| **Omitted study** \| **SMD** \| **95% CI** \| **P value** \| \| --- \| --- \| --- \| --- \| \| Jouve 2013 \| -0.11 \| -0.95, 0.74 \| 0.778 \| \| Renghi 2013 \| 0.01 \| -0.92, 0.94 \| 0.982 \| \| Zheng 2016 \| 0.01 \| -0.91, 0.94 \| 0.974 \| \| Bell 2019 \| -0.01 \| -0.94, 0.91 \| 0.974 \| \| Klotz 2020 \| -0.03 \| -0.94, 0.89 \| 0.947 \| \| Krige 2022 \| 0.01 \| -0.92, 0.94 \| 0.977 \| \| Chedgy 2023 \| 0.03 \| -0.90, 0.96 \| 0.944 \| \| Lee 2024 \| -0.03 \| -0.95, 0.88 \| 0.935 \| \| Opincans 2025 \| 0.31 \| 0.05, 0.58 \| **0.026** \| \|  \|  \|  \|  \| |
| **Urinary Retention** | **Mobilization** |
| \| **Omitted study** \| **RR** \| **95% CI** \| **P value** \| \| --- \| --- \| --- \| --- \| \| Almeida 2011 \| 0.37 \| 0.02, 6.41 \| 0.271 \| \| O'Neill 2012 \| 0.66 \| 0.27, 1.62 \| 0.187 \| \| Hughes 2015 \| 0.38 \| 0.02, 9.70 \| 0.331 \| \| Lee 2024 \| 0.27 \| 0.02, 3.82 \| 0.168 \| \|  \|  \|  \|  \| | \| **Omitted study** \| **SMD** \| **95% CI** \| **P value** \| \| --- \| --- \| --- \| --- \| \| Bell 2019 \| -1.57 \| -10.09, 6.95 \| 0.257 \| \| Calixto-Fl. 2020 \| -1.22 \| -13.81, 11.37 \| 0.435 \| \| Opincans 2025 \| -0.48 \| -4.40, 3.45 \| 0.366 \| \|  \|  \|  \|  \| |
| **Length of stay** |  |
| \| **Omitted study** \| **SMD** \| **95% CI** \| **P value** \| \| --- \| --- \| --- \| --- \| \| Renghi 2013 \| -0.14 \| -0.44, 0.16 \| 0.334 \| \| Ball 2016 \| -0.14 \| -0.44, 0.17 \| 0.338 \| \| Zheng 2016 \| -0.13 \| -0.44, 0.17 \| 0.348 \| \| Mungroop 2016 \| -0.15 \| -0.45, 0.15 \| 0.284 \| \| Bell 2019 \| -0.14 \| -0.44, 0.16 \| 0.314 \| \| Klotz 2020 \| -0.09 \| -0.38, 0.21 \| 0.537 \| \| Calixto-Fl. 2020 \| -0.07 \| -0.35, 0.20 \| 0.566 \| \| Gathege 2021 \| -0.12 \| -0.43, 0.18 \| 0.390 \| \| Krige 2022 \| -0.12 \| -0.43, 0.19 \| 0.401 \| \| Chedgy 2023 \| -0.13 \| -0.44, 0.18 \| 0.371 \| \| Lee 2024 \| -0.14 \| -0.45, 0.17 \| 0.332 \| \| Opincans 2025 \| 0.01 \| -0.14, 0.16 \| 0.880 \| |  |

**Appendix 9 Exploratory meta-regression analyses**

Exploratory meta-regression analyses were conducted using random-effects to investigate potential sources of heterogeneity. The following study-level moderators were assessed where feasible: surgical category (visceral vs non-visceral), incision type (laparotomy vs non-laparotomy), and publication year. Analyses were performed only for outcomes with a sufficient number of studies.

For **pain outcomes (k = 9 studies)**, no significant association was observed for surgical category (p = 0.662), incision type (p = 0.120), or publication year (p = 0.985). Residual heterogeneity remained high (I² = 98.3%).

For **opioid consumption (k = 7 studies)**, no significant association was observed for surgical category (p = 0.538), incision type (p = 0.810), or publication year (p = 0.541). Residual heterogeneity remained high (I² = 96.8%).

For **hypotension (k = 10 studies)**, no significant association was identified for surgical category (p = 0.376), incision type (p = 0.809), or publication year (p = 0.863), and none of the tested moderators explained between-study heterogeneity (all R² = 0%). Residual heterogeneity was moderate (I² = 35.9%).

For **bowel function (k = 9 studies)**, no significant association was observed for surgical category (p = 0.801), incision type (p = 0.906), or publication year (p = 0.146). Residual heterogeneity remained high (I² = 95.2%).

For **length of stay (k = 12 studies)**, no significant association was identified for surgical category (p = 0.579), incision type (p = 0.801), or publication year (p = 0.104).

Meta-regression analyses were not performed for the remaining outcomes due to the limited number of included studies (urinary retention: k = 4; mobilization: k = 3), rendering such analyses methodologically inappropriate and insufficiently powered.

**References**

1. Wan X, Wang W, Liu J, Tong T. Estimating the sample mean and standard deviation from the sample size, median, range and/or interquartile range. *BMC Med Res Methodol* 2014;**14**: 135.

2. Luo D, Wan X, Liu J, Tong T. Optimally estimating the sample mean from the sample size, median, mid-range, and/or mid-quartile range. *Stat Methods Med Res* 2018;**27**(6): 1785-1805.
